# Ovarian Cancer: In Search of Better Marker Systems Based on DNA Repair Defects

**DOI:** 10.3390/ijms14010640

**Published:** 2013-01-04

**Authors:** Dominic Varga, Miriam Deniz, Lukas Schwentner, Lisa Wiesmüller

**Affiliations:** Department of Gynecology and Obstetrics, University Ulm, Germany, Prittwitzstraße 43, 89075 Ulm, Germany; E-Mails: dominic.varga@uniklinik-ulm.de (D.V.); miriam.deniz@uniklinik-ulm.de (M.D.); lukas.schwentner@uniklinik-ulm.de (L.S.)

**Keywords:** ovarian cancer risk, early detection, DNA repair errors, functional biomarker, prediction of therapeutic responsiveness

## Abstract

Ovarian cancer is the fifth most common female cancer in the Western world, and the deadliest gynecological malignancy. The overall poor prognosis for ovarian cancer patients is a consequence of aggressive biological behavior and a lack of adequate diagnostic tools for early detection. In fact, approximately 70% of all patients with epithelial ovarian cancer are diagnosed at advanced tumor stages. These facts highlight a significant clinical need for reliable and accurate detection methods for ovarian cancer, especially for patients at high risk. Because CA125 has not achieved satisfactory sensitivity and specificity in detecting ovarian cancer, numerous efforts, including those based on single and combined molecule detection and “omics” approaches, have been made to identify new biomarkers. Intriguingly, more than 10% of all ovarian cancer cases are of familial origin. *BRCA1* and *BRCA2* germline mutations are the most common genetic defects underlying hereditary ovarian cancer, which is why ovarian cancer risk assessment in developed countries, aside from pedigree analysis, relies on genetic testing of *BRCA1* and *BRCA2*. Because not only *BRCA1* and *BRCA2* but also other susceptibility genes are tightly linked with ovarian cancer-specific DNA repair defects, another possible approach for defining susceptibility might be patient cell-based functional testing, a concept for which support came from a recent case-control study. This principle would be applicable to risk assessment and the prediction of responsiveness to conventional regimens involving platinum-based drugs and targeted therapies involving poly (ADP-ribose) polymerase (PARP) inhibitors.

## 1. Introduction

**Figure 1:**
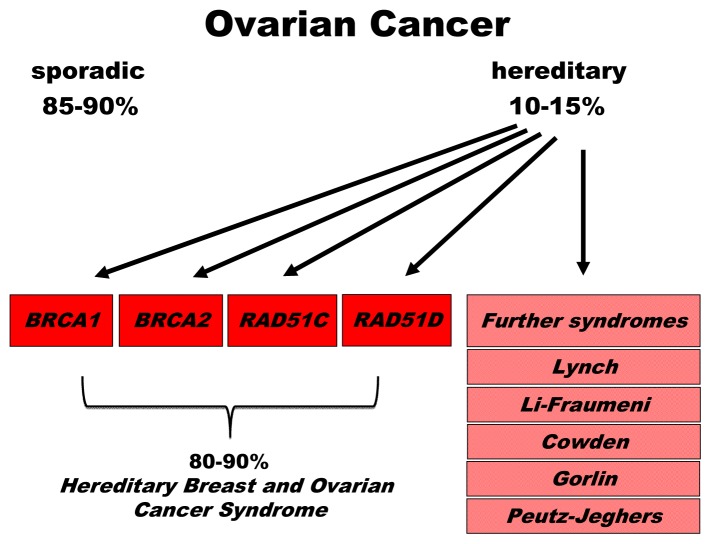
Schematic overview of susceptibility genes for familial ovarian cancer. Ten to fifteen percent of ovarian cancer cases are of familial origin. Until now, 16 susceptibility genes causing at least six cancer susceptibility syndromes have been identified [12,13]. However, approximately 80 to 90% of the hereditary ovarian cancer cases can be explained by mutations in *BRCA1*, *BRCA2*, *RAD51C*, and *RAD51D*, which cause Hereditary Breast and Ovarian Cancer Syndrome.

Ovarian cancer is mostly a disease of postmenopausal women [1]. The mean age of ovarian cancer diagnosis is approximately 69 years, and 80% of all ovarian cancer cases are diagnosed after the age of 50. The lifetime risk for ovarian cancer accumulates to approximately 1.5%. According to the American Cancer Society, the projected incidence and death rates of this disease in the US are approximately 22,000 and 15,000, respectively, which means that ovarian cancer accounts for 3% of all female cancers and represents the most fatal gynecological malignancy [2]. Although reproductive, demographic, and lifestyle factors affect the risk of ovarian cancer, the single greatest ovarian cancer risk factor is family history of the disease [3]. Hereditary ovarian cancer comprises 10 to 15% of all cases of ovarian malignancies and is mainly associated with germline mutations in the *BRCA1* and *BRCA2* genes [4,5]. Consequently, these two high-risk genes have become an integral part of genetic testing programs worldwide. Following more recent discoveries of susceptibility genes for ovarian cancer, the molecular diagnosis of ovarian cancer predisposition within risk families was extended to novel genes such as *RAD51C* [6] (see Table 1 and Figure 1).

Regarding its histology, ovarian cancer is a heterogeneous disease with surface epithelial ovarian cancer (as compared with germ or sex cord-stromal tumors) representing the most common form, particularly in hereditary cases [14]. Five major subtypes among epithelial tumors, papillary serous, endometrioid, mucinous, clear cell, and transitional cell can be differentiated, and certain predisposing factors promote the appearance of certain subtypes. Regarding its pathogenesis, a low-grade, multi-step pathway generates benign and borderline up to malignant forms of the cancer. However, a high-grade pathway underlies the formation of rapidly growing, undifferentiated, and highly aggressive tumors [12]. This pathway dominates among epithelial ovarian cancers and is further promoted in *BRCA1-* and *BRCA2*-mutated tumors, thus necessitating major research efforts to improve diagnosis, as early detection is of utmost importance for therapeutic success.

In addition to clinical examination, an ultrasound of the pelvis, followed by serum cancer antigen 125 (CA125) tumor marker determinations are the most frequently used screening examinations for ovarian cancer. Various interpretation criteria exist, but sensitivities are not satisfactory [15,16]. In 2008, the US Preventive Services Task Force (USPSTF) issued a statement on “fair evidence” that ovarian cancer screening (consisting of ultrasound and CA125 tumor marker determination) had little effect on reducing mortality rates, while instead increasing the risk of harm (diagnostic operations, decline in quality of life). Computerized axial tomography (CAT) scan and nuclear magnetic resonance imaging (MRI) are techniques applied for preoperative evaluation of a suspected ovarian malignancy and for surgical planning, but not as primary screening tools [17].

Today, neither early detection nor therapy of ovarian cancer is effective. Due to its biological aggressiveness, new strategies, especially in defining persons at risk, are necessary. A conceivable approach may address DNA repair dysfunction as a potential common denominator because there are phenotypic and molecular similarities between hereditary ovarian tumors with mutations in high risk susceptibility and DNA repair genes compared with some sporadic tumors, which display a high response rate to platinum agents, high-grade, serous histology, *BRCA* mutations in the tumor and similar molecular abnormalities [18].

## 2. Genetics of Hereditary Cancer

### 2.1. *BRCA1* and *BRCA2*

Compared with 5 to 10% of breast cancer cases, 10 to 15% of ovarian cancer cases are caused by a hereditary predisposition [5,19]. Until the recent discoveries of *RAD51C* and *RAD51D* [6,7], the most notable genes contributing to the 4.6-fold relative risk conferred by hereditary ovarian cancer susceptibility had been *BRCA1* and *BRCA2*. Deleterious mutations in *BRCA1* lead to a lifetime ovarian cancer risk of approximately 20%–50% and in *BRCA2* of approximately 10%–20% (see Table 1). In support of a particularly severe influence of *BRCA1* mutations, it was further shown that the mean age at diagnosis of ovarian carcinoma is significantly younger in *BRCA1* (49) *versus BRCA2* (58) mutation carriers but still significantly reduced compared with the general population (68) [6]. In terms of histological features, ovarian cancer from both *BRCA1* and *BRCA2* mutation carriers were found to predominantly belong to the high-grade serous carcinoma subtype [20,21].

*BRCA1* and *BRCA2* are of utmost importance in DNA repair, cell cycle checkpoint control, and maintenance of genomic stability [4]. According to Kinzler and Vogelstein’s definition [22], both of these genes belong to the group of caretakers. Compared with a gatekeeper, a caretaker is not directly involved in tumor initiation or promotion, but rather, its involvement is indirect. Thus, the inactivation of a caretaker leads to genomic instability including mutations in oncogenes and tumor suppressor genes, thereby disabling cell death and cell cycle checkpoint functions and enabling tumor growth.

### 2.2. Susceptibility Genes with Involvement in the *BRCA*-Fanconi Anemia Pathway of DNA Repair

**Figure 2:**
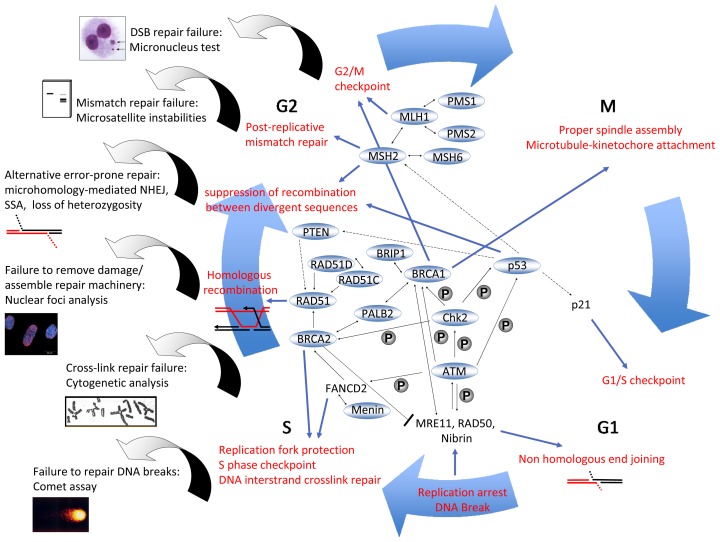
Interactome of ovarian cancer susceptibility gene products summarizing DNA damage response activities and assays for the detection of functional defects. Functional and physical interactions between DNA repair-related ovarian cancer susceptibility gene products are schematically drawn and their role in different DNA repair mechanisms and checkpoint responses during the cell cycle are indicated [25–35]. Various readouts for DNA repair failure that have been assayed as potential biomarkers for ovarian cancer risk are positioned next to the corresponding mechanisms, as discussed in the text [12,24,36–39]. One-headed arrow, recruitment or activation; two-headed arrow, physical interaction; stippled arrow, transcriptional regulation; encircled “P”, phosphorylation; blocked line, inhibition; blue-circled protein names, ovarian carcinoma susceptibility gene product; red letters, processes with relevance for genome stability; vaulted black arrow, detection of a repair defect. Note that breaks may also occur in cell cycle phases other than G1/S phase.

Ten years after the discovery of *BRCA2* as a breast and ovarian cancer susceptibility gene, it was found to be the same gene as *FancD1*, *i.e.*, one of the genes causing the childhood disease Fanconi anemia (FA), upon bi-allelic mutation [23]. FA patients suffer from bone marrow failure, skeletal abnormalities, reduced fertility, genomic instability, and cancer predisposition [24]. FA can be diagnosed through cytogenetic analysis of peripheral blood lymphocytes, as patients’ cells fail to repair damage resulting from *ex vivo* DNA cross-linker treatment (Figure 2).

After *BRCA2/FancD1*, two other genes, namely, *BRIP1*/*FancJ* and *PALB2*/*FancN*, were described as FA and breast and ovarian cancer susceptibility genes, suggesting a strong genetic connection between FA and hereditary breast and ovarian cancer [25,40]. BRIP1 encodes a helicase, also called BACH1, which was originally identified as a BRCA1 binding protein. PALB2 protein is also known to interact with BRCA2, facilitating BRCA2-mediated DNA repair by promoting the localization and stability of BRCA2. *BRIP1* and *PALB2* mutations are known to confer a two-fold increased risk of familial breast cancer. The precise relative risk for ovarian cancer has remained unclear.

The identification of a series of genes that are involved in the BRCA-FA DNA repair pathway inspired more systematic searches for other pathway components, which resulted in the discovery of two further prominent ovarian cancer risk genes in 2011 and 2012: *RAD51C* and *RAD51D* [6,7]. First, highly penetrant mutations were identified in the *RAD51C* gene in families with both breast and ovarian cancer (1.3%) but not in families with breast cancer only [6] (Table 1). As with *BRCA2/FancD1*, *BRIP1*/*FancJ*, and *PALB2*/*FancN*, bi-allelic mutations in *RAD51C*/*FancO* cause FA, and mono-allelic mutations increase the risk of breast and ovarian cancer. *RAD51C* families showed a striking complete segregation regarding gene mutation and the development of malignancies. More recently, *RAD51D* was found to be associated with ovarian cancer, but not breast cancer, with an estimated relative risk of 6.3 [7]. The encoded proteins RAD51C and RAD51D play roles in damage signaling, the recruitment of the recombinase RAD51 to the sites of damage, and processing repair intermediates in different complexes with products of other members of the RAD51 family of related genes, namely, RAD51B, XRCC2, and XRCC3 [41].

### 2.3. Further DNA Repair Genes and Syndromes Associated with Hereditary Ovarian Cancer

Aside from the above listed genes that have been linked with the BRCA-FA DNA repair pathway, other genes and genetic syndromes were also found to be associated with increased ovarian cancer risk. Mutations of specific genes including, but not limited to, MLH1, MSH2, MSH6, PMS1, and PMS2 are responsible for Lynch syndrome, which is associated with susceptibility to carcinomas of the ovary and endometrium [12,42]. In addition to mutations, epigenetic hypermethylation, and loss of mismatch repair (MMR) gene expression were observed in ovarian cancers. Ovarian cancer in Lynch syndrome patients is characterized by distinct histology, namely, the formation of endometrioid and related clear cell types rather than serous epithelial carcinomas. Nevertheless, Lynch syndrome also increases the risk of colorectal cancer and cancers of the stomach, small intestine, liver, bile duct, urinary tract, brain, and central nervous system, as well as possibly breast cancer. All the genes linked to Lynch syndrome play important roles in MMR, suggesting a malignant transformation through loss of this specific DNA repair activity. Mutations in MMR genes can be comprehensively detected by PCR-based analysis of microsatellite instabilities (MSI), which has been used for diagnostic purposes (Figure 2). From these observations, ovarian cancer risk is associated with a single additional defective DNA repair mechanism. At first sight, the functional defects in the BRCA-FA pathway and Lynch syndrome patients appear to be completely unrelated. However, mispairings can arise not only after DNA replication, but also after the pairing of divergent sequences during homologous recombination. Mispairings can reduce the accuracy of this fairly safe repair mechanism and lead to mutagenic events and carcinogenesis. Indeed, several groups demonstrated that MMR proteins also proofread homologous recombination processes in a manner that depends on, or acts independently of, classical MMR enzyme functions [26,27]. According to these studies, MutS homologs block heteroduplex extension when involved in mispairings, and MutL homologs stimulate MutS protein function, possibly destabilizing blocked intermediates, and triggering ATM and ATR signaling.

ATM and ATR belong to a protein family known as the phosphatidylinositol 3-kinase (PI3K)-related protein kinases and initiate a signaling cascade in response to DNA double-strand breaks (DSBs) and stalled replication forks, respectively, through the phosphorylation of many DNA repair proteins such as p53, BRCA1, Nibrin, and Chk2 [28]. Heterozygous carriers of Ataxia telangiectasia (AT)-causing mutations in the *ATM* gene have been associated with hereditary breast and ovarian cancer [43]. AT is a neurodegenerative, autosomal recessive disorder characterized by high sensitivity to ionizing radiation, immune deficiency, and cancer predisposition, especially for leukemia and lymphoma.

In this context, it is important to mention Li-Fraumeni syndrome (LFS), which is linked with germline mutations within the *TP53* gene. LFS shows autosomal dominant inheritance, as would be expected from the dominant-negative or even gain-of-function features of cancer-related p53 mutant proteins [44]. LFS is accompanied by a higher risk for developing osteosarcoma, soft tissue sarcoma, leukemia, and breast, brain, and ovarian cancer [45]. LFS is rare, but a recent analysis of breast cancer cases revealed that an unexpectedly high fraction of early-onset patients may have *TP53* mutations [46]. *TP53* mutations are found not only in LFS but also in 50 to 60% of high-grade serous ovarian carcinomas and may represent early events accompanied by DNA damage during high-grade pathogenesis [12]. p53 is known to exhibit crucial checkpoint functions in response to DNA damage, which may explain the high frequency of *TP53* mutations in *BRCA1*-mutated cells. Intriguingly, accumulating evidence in recent years has further demonstrated that p53 also controls homologous recombination independent of its checkpoint functions in a manner that is highly reminiscent of the proofreader activities of MMR proteins [29,47].

Mutations in *PTEN* cause the Cowden syndrome known as multiple hamartoma syndrome, which is associated with benign and malignant tumors of the breast and ovaries, in the latter case with the endometrioid type of ovarian carcinomas [12]. PTEN encodes a lipid phosphatase that functions as a tumor suppressor and is linked with the pathways involved with p53, RAS, and mTOR. It came as a great surprise when PTEN was shown to play an essential role in chromosomal stability through the induction of RAD51 and homologous recombination [48].

Ovarian cancer has also been associated with basal cell nevus (Gorlin) syndrome and multiple endocrine neoplasia type 1 and inherited breast–ovarian cancer syndromes. The protein Menin that is encoded by the gene *MEN1*, which is mutated in Gorlin syndrome, binds FancD2 and mediates DNA cross-link repair and homologous recombination [49–51].

In contrast to all the ovarian susceptibility genes and syndromes listed above, Peutz-Jeghers syndrome (PJS) has thus far not been linked with DNA repair. PJS is caused by a specific genetic mutation in the *STK11* gene and is associated with multiple polyps in the digestive tract that become noncancerous tumors. In addition to an increased risk of ovarian cancer, PJF also raises the risk of breast, uterine, and lung cancer [52]. *STK11* codes for a serine/threonine kinase that inhibits cellular proliferation, controls cell polarity, and interacts with the mTOR pathway.

## 3. DNA Repair Mechanisms

The genetic defects listed above, which predispose a patient to familial ovarian cancer, revealed that, with one single exception (*STK11*), each single ovarian cancer susceptibility gene can be directly or indirectly linked with DNA repair, particularly with the BRCA-Fanconi Anemia and mismatch repair pathways. Therefore, DNA repair dysfunction may represent a common denominator of this type of cancer. The precise knowledge of the molecular details of these mechanisms may help to identify novel diagnostic, predictive biomarkers, and targets for the development of novel therapies.

DNA suffers from a wide range of lesions, e.g., DSBs, single-strand breaks, intrastrand, and interstrand cross-links, free radical oxidative damage, or incorporation of mismatched bases due to errors in replication. DSBs are among the most toxic and lethal DNA lesions [53] and can be caused by exogenous sources such as ionizing radiation. Additionally, DSBs arise endogenously, e.g., as a result of replicative stress [54]. If left unrepaired, DNA lesions can result in permanent cell cycle arrest or cell death.

DNA repair pathways are determined by the type of damage and the cell cycle stage at which it arises (Figure 2). If one strand is injured and the complementary strand is still intact and can be used as a template, base excision repair, nucleotide-excision repair, or mismatch repair are employed to restore the lesion. Base excision repair is dedicated to removing smaller adducts such as oxidative base modifications [55]. In base excision repair, DNA glycosylases remove the incorrect or modified bases, apurinic/apyrimidinic endonuclease subsequently incises the sugar-phosphate backbone next to the missing base; the gap is then filled and the DNA sealed. PARP1 is a key regulator of this repair pathway, as it assembles base excision repair components such as X-ray repair cross-complementing protein 1 (XRCC1) [56]. Upon activation by nicked, gapped or broken DNA, PARP1 cleaves NAD^+^ to nicotinamide and ADP-ribose and generates long branched polymers of ADP-ribose on PARP itself or on other DNA repair proteins and chromatin components, thereby modifying the functions of these proteins.

DNA mismatch repair (MMR), which repairs single-base mispairings or larger insertion/deletion loops, represents another repair mechanism of interest in the context of ovarian cancer risk [12]. Mispaired bases and single base insertion/deletion loops are recognized by the MutSβ complex consisting of the proteins MSH2 and MSH6 [55]. The MutSβ or MSH2–MSH3 complex detects larger insertion/deletion loops. Mismatch detection is followed by ternary complex formation with MutL complexes composed of MLH1 and PMS2, PMS1, or MLH3. The following incision step was reported to be supervised by PARP1 through interactions with MutSα and exonuclease 1, thereby assuring that it is limited to sites of mispairings [57]. Excision of the strand circumventing the mismatch, repair synthesis, and ligation complete this repair pathway.

For the most problematic incident of a DSB, homologous recombination, nonhomologous end-joining (NHEJ), or single-strand annealing (SSA) are the repair pathways of choice because there is no complementary strand that can be used for repair [58]. The homologous recombination pathway is the most accurate DSB repair mechanism, in which a homologous stretch of DNA on a sister chromatid serves as template to guide the repair of the broken strand. NHEJ is a less accurate form of DSB repair, as it frequently (canonical NHEJ) or always (microhomology-mediated) results in the loss or gain of nucleotides and, hence, in the disruption of genomic integrity. Similarly, SSA starts with DNA end processing, however, it is followed by DNA annealing between genomic repeat sequences with extensive homologies. SSA always leads to the loss of the intervening DNA sequences, and therefore, is considered mutagenic. The homologous template necessary for homologous recombination is only present during the S and G2 phases of the cell cycle, thus limiting homologous recombination to these cell cycle phases, whereas NHEJ can also take place during the G1 phase [58]. Repair of DNA cross-links initiates at stalled replication forks, involves homologous recombination, and takes place during the S phase [30].

Canonical NHEJ essentially utilizes the V(D)J recombination machinery and is mostly relevant regarding diseases of the hematopoietic system [59]. DSB repair other than canonical NHEJ is initiated by the recruitment of the MRN complex to the DNA lesion. MRN, composed of the heterotrimer MRE11-RAD50-Nibrin, catalyzes the processing of DNA ends, whereby extended single-stranded DNA overhang formation for homologous recombination and SSA require the recruitment of the exonuclease CtIP by BRCA1 [58]. The resulting 3′ single-stranded ends are rapidly coated by the replication protein A (RPA), which in turn is replaced by RAD51 through action of BRCA1, BRCA2, PALB2, and auxiliary proteins including RAD51C and RAD51D [41,60]. The newly formed RAD51 nucleoprotein filaments then invade the sister chromatid and initiate the homology search and repair synthesis. From that point on, DSBs can be repaired by classical DSB repair or alternative synthesis-dependent strand annealing. The classical model involves Holliday Junction formation and its resolution in a non-crossover or a crossover manner, a process again involving RAD51C and RAD51D, whereas during synthesis-dependent strand annealing, the displacement of the resynthesized strand and reannealing with the parental strand reconstitute the intact duplex [41,58]. Compared with homologous recombination, the mutagenic pathways SSA and microhomology-mediated NHEJ are mechanistically less well defined; however, they are clearly independent of the central invasion and strand exchange steps mediated by RAD51 and its auxiliary factors. Interestingly, PARP1 has been identified as a component of the microhomology-mediated NHEJ pathway [61].

DSB repair and the homologous recombination pathway, in particular, are of highest relevance in the context of ovarian tumor formation and its treatment [41,60]. Thus, the respective repair genes include risk genes for ovarian and breast cancer, especially *BRCA1*, *BRCA2*, and *RAD51C*. In addition, in non-tumor cells, DNA repair and the accuracy of DNA repair are also controlled by DNA damage checkpoints that halt cell cycle progression at the G1/S and G2/M boundaries (Figure 2). *ATM*, which is mutated in Ataxia telangiectasia patients and in family members with increased breast and ovarian cancer, encodes a kinase that senses DSBs through the MRN complex and triggers a cascade of phosphorylation events, implementing high-fidelity DNA repair and checkpoint control [28,43]. Regarding potential targeted therapies for ovarian cancer patients in the future, it is important to consider that PARP1 was reported not only to promote base excision repair and microhomology-mediated NHEJ but also to play a role in the detection of stalled replication forks, namely, in the recruitment of the MRN complex for end processing, and also in homologous recombination to restart the stalled replication fork [62].

## 4. Diagnostics: Present and Future

### 4.1. Pathology of Ovarian Cancer

Epithelial ovarian cancer is a heterogeneous disease characterized by diverse subtypes, which are distinguished by histological, clinical, and molecular features and are relevant in the context of biomarker development [12]. Germline *BRCA1* and *BRCA2* mutations are predominantly associated with high-grade serous carcinoma formation [20,21] (Table 2). The serous histotype represents the most common type of ovarian cancer (70%) and is sub-classified into low- or high-grade cancer [63]. Clinicopathological data suggest that low- and high-grade serous carcinomas have different genetic origins or are the result of different genetic defects in different pathways. *TP53* mutations (≥50%), and, to a lesser extent, possibly *HER2*/*neu* and *AKT2* gene amplifications, are genetic alterations that are associated with high-grade serous carcinomas, whereas *BRAF* and *KRAS* mutations are found in two-thirds of low-grade serous carcinomas [64]. Another histological subtype comprising 10 to 20% of epithelial cancers is endometrioid carcinoma, which is often associated with endometriosis and endometrial cancer of the uterus [65]. *PTEN* mutations, *β-catenin* gene mutations or alternative deregulation of these pathways and microsatellite instabilities due to loss of expression of MLH1, MSH2, or other MMR genes have each been reported in up to 20% of endometrioid ovarian carcinomas and are also observed in the closely related endometrioid endometrial cancer [65,66]. High-grade carcinomas of FIGO grade 2 and 3 frequently also carry *TP53* mutations.

Mucinous and clear cell tumors represent additional clinically important subtypes of ovarian cancer, but, due to their rarity, have been less well characterized in molecular terms. Ovarian clear cell carcinoma accounts for approximately 5% of all ovarian cancer cases and is often diagnosed at an early stage. Clear cell carcinomas share molecular features with endometrioid ovarian carcinomas because microsatellite instability can be observed in a subset and *PTEN* mutations at a low frequency [65,68]. Mucinous tumors are classified either as benign or borderline in some instances or as malignant in others; however, pathological classification is difficult for this heterogeneous type of tumor. *KRAS* gene mutations (>75%) appear to play a critical role in the etiology of mucinous ovarian cancer [69]. Additionally, expression profiling has revealed the differential expression of genes involved in signal transduction, cell cycle regulation, and cytoskeleton regulation beyond the characteristic mucin gene overexpression [70].

In conclusion, each subtype appears to carry characteristic defects in certain pathways, which suggests that current clinical management is inappropriate. Moreover, even within the most common histological subtypes, the molecular pathogenesis of low-grade *versus* high-grade tumors appears to be largely distinct. One approach to cope with this heterogeneity was to generate a model for the development of type I and II tumors [64]. Type I tumors include low-grade serous carcinoma, low-grade endometrioid carcinoma, mucinous carcinoma, and a subset of clear cell carcinomas, which develop in a stepwise fashion from well-recognized precursors, namely, in most cases, borderline tumors. Most type I tumors are slow growing. Type II carcinomas include high-grade serous carcinoma, high-grade endometrioid carcinoma, undifferentiated carcinoma, possibly certain clear cell carcinomas, and malignant mixed mesodermal tumor (carcinosarcoma). Type I tumors often harbor somatic mutations of genes that encode protein kinases, including *BRAF*, *KRAS*, and other signaling molecules such as PTEN and ß-catenin. Type II tumors are characterized by a high frequency of *TP53* mutations, fast growth, and chromosomal instability.

### 4.2. State-of-the-Art and Recent Efforts to Identify Single Molecule or Combined Biomarkers

An ultrasound of the pelvis is the most frequently used screening examination for ovarian cancer, aside from abdominal examination [15]. Despite the existence of various interpretation criteria, a lack of ultrasound standardization is pervasive. Exploratory laparoscopy or laparotomy, CAT scan, and MRI are techniques applied to determine the cause of a patient’s symptoms or to establish tumor characteristics, but not for screening purposes [17]. In addition to the aforementioned diagnostic tools, CA125 has been used as a serum marker [71]. It is widely accepted that CA125 is a useful preoperative marker to predict malignant potential. However, it is also known that CA125 is not useful as a screening marker [15]. CA125 is correlated with a poor outcome because high levels of CA125 are associated with larger tumor masses. Conversely, CA125 is of no use for the detection of early onset ovarian cancer, as only 50% of early-stage I and II cases demonstrate elevated CA125 levels. Moreover, many benign ovarian and non-gynecological incidents are also associated with an increase in CA125, particularly in patients with underlying endometriosis [72].

Another potential diagnostic marker, which generated enthusiasm at the beginning of the millennium, is osteopontin. Osteopontin was initially found to be differentially expressed during a cDNA microarray study of RNA isolated from ovarian cancer cell lines and human ovarian surface epithelial cells [73]. More recently, research has focused on kallikreins as potential biomarkers [71]. In two studies published in 2003 [74,75], the majority of the screened ovarian cancer patients showed elevated human kallikrein 6 and 8 serum levels, respectively, whereas elevated levels of kallikrein were not detected in the controls. At this stage, the evaluation of its predictive power would require validation in large-scale studies. High preoperative plasma bikunin levels have been reported to represent a strong and independent favorable prognostic marker for ovarian cancer [76]. In a DNA profiling study of primary serous ovarian and fallopian tube carcinomas, the amplified genes included *SPINT2*, *i.e.*, the gene encoding Bikunin [77]. In addition to these candidate markers, multiple angiogenic factors and cytokines have been evaluated because a high degree of tumor angiogenesis has been shown to correlate with poor survival in women with ovarian cancer [78]. Among these angiogenic factors, VEGF appears to be a promising prognostic factor for ovarian cancer, but does not appear to be useful for screening.

Candidate molecules have been evaluated as single markers or in combination with CA125. From these studies, the human epididymis protein 4 (HE4) turned out to be the most promising candidate for the detection of ovarian cancer [79,80]. Compared with CA125, HE4 showed lower sensitivity, but it did improve specificity in detecting malignant *versus* benign cases [79–83]. However, there is conflicting data regarding the predictive value of HE4 as a marker of malignant adnexal mass [84,85]. In particular, the authors of a recent meta-analysis concluded that HE4 is not superior to CA125 in predicting epithelial ovarian cancer, whereas the risk of ovarian malignancy algorithm (ROMA) was found to be a promising predictor to replace CA125 [86]. Nevertheless, evidence has accumulated indicating that high preoperative HE4 levels are significantly associated with an unfavorable prognosis in ovarian cancer patients and significantly associated with risk of an incomplete tumor resection [87,88]. Importantly, evidence for HE4 as an independent predictor of ovarian cancer relied on serum measures [87,88], whereas immunohistochemical expression of HE4 has been found in benign, borderline, and malignant ovarian tumors [89]. Altogether, there are controversial results of the impact of HE4 in predicting adnexal masses, but it appears to be associated with an inferior outcome. Most important, compared with CA125, HE4 exhibited a considerable capacity to distinguish between ovarian cancer and endometriosis [82]. Thus, even though CA125 has not achieved satisfactory sensitivity and specificity in detecting ovarian cancer as a single predictive and prognostic marker, combined clinical use of CA125 with HE4 may significantly improve their predictive values [83,90]. Consequently, HE4 has been approved for clinical use along with CA125 to predict epithelial ovarian cancer with a pelvic mass or in remission after chemotherapy. However, because of the strong effect of age on HE4, thresholds for HE4 should be defined for specific ages [91].

### 4.3. “Omics”-Based Approaches to Identify Potential Biomarkers

In the last two decades, more than 200 biomarkers for ovarian cancer have been proposed, but only a few of them are suitable for clinical routine. A biomarker search focused on detection and diagnosis in the beginning, but more recently shifted to prognostic markers for personalized treatment and risk stratification [92]. Most of these studies engaged omics technologies, *i.e.*, mostly proteomics as well as genomic and epigenomic analyses, and revealed molecular changes associated with the tumorigenesis, pathophysiology, and outcome of ovarian cancer.

#### 4.3.1. Protein Markers

Proteomic profiling mostly relies on two-dimensional gel electrophoresis and mass spectrometry, whose sensitivity is limited. More recently, it has utilized antibody arrays, which are limited by the availability of immunoassays [80,93]. Since the 1990s, more than 160 proteins have been reported to be differently expressed in early ovarian cancer patients compared with healthy women (reviewed in [94]). Nolen and Lokshin [94] postulated that most of these potential biomarkers from body fluids can be classified into six functional subgroups that reflect the different biological pathways of ovarian tumorigenesis: mediators of inflammation/cytokines/chemokines (e.g., TNFα), acute-phase reactants/coagulation factors, adhesion molecules/proteases (e.g., CA125), hormones/related molecules (e.g., leptin), and growth/angiogenesis factors (e.g., VEGF). Additionally, groups of proteins involved in apoptosis and energy homeostasis and of undefined function, including HE4, have been identified. Le Page and colleagues [95] categorized biomarkers for chemoresistance/survival identified by immunohistochemistry into nine main groups: oncogenes and tumor suppressors such as p53; proliferation markers such as Ki67; cell cycle regulators such as cyclins; apoptosis modulatory proteins such as CD95 or Bcl-2 family members; DNA repair enzymes such as BRCA1, BRCA2, and PARP1; markers of angiogenesis such as VEGF; immunological factors such as cytokines; tyrosine kinase receptors such as EGFR: and, cadherins/β-catenin. Presently, multimarker panels containing different combinations of known protein biomarkers are under evaluation. A promising panel under the trade name OvaSure™ (Labcorp, NC, USA) monitors the serum biomarkers leptin, prolactin, osteopontin, insulin-like growth factor II, and macrophage inhibitory factor combined with CA125 and was reported to perform with a sensitivity of 95.3% and a specificity of 99.4% [96]. However, a few months after the report, Macintosh and colleagues [97] noted severe statistical errors. Consequently, the US Food and Drug Administration intervened, and the OvaSure^TM^ test was withdrawn from the market. Several other groups investigated similar panels for detecting ovarian cancer in an early stage with sensitivities and specificities ≥90%, but prospective randomized studies are necessary for clinical implementation [94,98–100].

#### 4.3.2. Patterns of Genetic and mRNA Expression Changes

As has been observed with other cancer entities, genetic alterations in ovarian cancer are associated with clinical characteristics. Ovarian cancer is thought to develop *de novo* either from the epithelium of the ovarian or the fallopian tube surface, whereby malignant transformation involves a multi-step process with the accumulation of genetic lesions [12]. Because of the existence of increasingly higher resolution techniques, today, genetic alterations can be identified as so-called copy number variations (CNVs), *i.e.*, changes in genomic copy number involving DNA fragments as short as 50 bp [101,102]. These alterations include quantitative divergences such as repeats, duplications, and deletions. CNVs are thought to account for 13% of the human genome and appear to encompass more nucleotides and arise more frequently than single nucleotide polymorphisms (SNPs) [103]. CNVs can affect gene expression by altering gene dosage and influencing neighboring regulatory regions [104]. Copy number aberrations (CNAs), a sub-class of CNVs that arise somatically, are mostly found in tumor DNA, reflecting genomic instabilities developed during tumorigenesis, and are hallmarks of cancer-related gene deregulation [105].

CNAs are most frequently found in the DNA of solid tumors and show a wide range in number and types of alterations [105]. Mutations in genes that are related to DNA repair and were identified in cancer-prone syndromes such as *BRCA1*/*2* (hereditary breast and ovarian cancer) or *TP53* (LFS) are known to be associated with chromosomal instability [106,107]. In this context, it is important to note that during the last years, in addition to single-nucleotide mutations, CNAs were also identified in *BRCA1* and *BRCA2* through multiplex ligation-dependent probe amplification (MLPA). Thus, a large-scale MLPA exploration of 1506 German families for pathogenic genomic rearrangements in the *BRCA1* gene and of 450 families for gross rearrangements in *BRCA2* showed that in high-risk groups for hereditary breast and ovarian cancer, the prevalence of rearrangements was 2.1% [108]. Although only a minority of ovarian cancer patients have somatic *BRCA1* and *BRCA2* mutations [109], Hilton and colleagues [110] reported that BRCA1 and/or BRCA2 functions were found to be compromised in the majority of both hereditary and sporadic ovarian cancer cases through a combination of gene alterations including mutations, the loss of heterozygosity, epigenetic silencing mechanisms, and unknown mechanisms leading to a lack of mRNA. From their results, the authors concluded that BRCA1 and/or BRCA2 dysfunction may be of nearly universal importance for the process of ovarian carcinogenesis. The *TP53* gene is mutated in 50 to 80% of high-grade invasive ovarian carcinomas but rarely in other ovarian cancer subtypes including borderline serous tumors [12]. For comparison, *KRAS* and *BRAF* mutations are rarely detected in high-grade invasive carcinomas but are often present in borderline ovarian tumors, low-grade adenocarcinomas, and adjacent benign epithelium. Independent of the tumor grade, single-nucleotide mutations, and CNAs additionally target the PI3K/AKT signaling pathway in ovarian cancer, including the susceptibility gene *PTEN* [111].

Analogous to corresponding studies on breast cancer genomes, analyses of CNAs were performed to categorize ovarian cancers into distinct subtypes. Engler *et al*. [112] evaluated 72 high-grade serous ovarian carcinomas and estimated that there are two primary subgroups characterized by a distinct CNA cluster. Regions of frequently increased CNAs were localized on 1q, 3q26, 7q32-q36, 8q24, 17q32, and 20q13, whereas regions of decreased CNAs were localized on 1p36, 4q, 13p, 16q, 18q, and X12. The genes reproducibly targeted by gains or losses were *MYCL1*, *EVI1*, *BRAF*, *MYC*, *KRAS*, *CCNE1*, *TP73*, *RB1*, and *MN1* [69]. The subgroups differed at eight genomic regions along 8p21.3, 8p23.2, 12p12.1, 17p11.2, 19q12, 20q11.21, and 20q13.2 and in progression-free and in overall survival. Interestingly, positive correlations were observed between the different loci of frequent CNAs such as between gains on 19 and 20q and gain on 20q and loss of X, which is also associated with patient outcome [113].

To better define the molecular subgroups associated with clinical features, mRNA expression profiling-based studies have been performed. In this way, it was found that mucinous and clear cell ovarian carcinomas can be distinguished from serous subtypes independent of tumor stage and grade [70,114]. Expression profiling of serous tumors of the ovary showed that the majority of the low-grade tumors clustered with the low malignant tumors, whereas the high-grade tumors were characterized by the enhanced expression of genes linked to cell proliferation and chromosomal instability [115]. A widely recognized study by Tothill and colleagues [116] identified six molecular subtypes of ovarian cancer through microarray gene expression profiling due to a comparatively large number of cases, namely, 285 serous and endometrioid tumors. Importantly, these molecular subtypes reflected histopathologically relevant subtypes, namely, serous low malignant, low-grade endometrioid, and four high-grade serous and endometrioid subtypes, and were associated with distinct survival characteristics. Additionally, gene expression profiling was found to discriminate between primary chemoresistant and primary chemosensitive ovarian cancers and to predict overall survival and relapse-free survival [117,118]. To predict overall, relapse-free, and progression-free survival after platinum-based chemotherapy, Kang *et al*. [119] produced a score via the expression profiling of known genes involved in DNA repair after corresponding treatment. Patients with improved survival showed a high score and *vice versa*. From their data, the authors postulated that this score may help to determine patient benefit before the initiation of the first-line therapy, as patients in the lowest scoring category appeared to benefit from alternative therapies rather than platinum-based regimes [119].

Taken together, there are numerous studies trying to refine the molecular understanding of ovarian carcinomas via array-based analysis. In recent years, the similarities between expression patterns from different studies including stromal response/extracellular matrix, immune, and proliferation signatures and between gene sets predicting the survival or platinum responses have been observed. Thus, the identification of molecular subsets from expression studies has helped to better understand the biology and pathogenesis of ovarian cancer and represents a promising marker for the clinical categorization of ovarian cancer, either alone or in combination with genomic profiling and/or functional marker sets.

#### 4.3.3. Epigenetic Markers

One major challenge in the treatment of relapsed ovarian cancer is the acquisition of drug resistance after first-line chemotherapy with platinum-based drugs. Several mechanisms have been discussed for resistance development such as genetic alterations in DNA repair genes and genes involved in apoptosis, cell cycle control, or drug uptake mechanisms (summarized in [120]). Epigenetic deregulation, caused by aberrant DNA methylation, histone modification, or RNA interference, is frequent in ovarian cancer. It has been investigated for single loci and in the gene panel format up to the microarray-based methylome scale [121]. From these studies, Balch *et al*. [121] listed more than 100 potentially prognostic biomarkers such as hyper- and hypomethylated genes and misexpressed or misprocessed microRNAs, which are associated with the detection of ovarian cancer, tumor stage and grade, chemoresponse, or survival. One very well-established epigenetic mechanism is the aberrant DNA hypermethylation of CpG islands in the promoter region of tumor suppressor genes that result in gene silencing [122]. The DNA repair genes *BRCA1*, *MLH1*, and *MGMT* were among those genes affected by hypermethylation in ovarian cancer [123–126]. One study, investigating 50 patients *versus* 40 controls, demonstrated an association between ovarian cancer and gene hypermethylation in at least one of six genes, including *BRCA1* [127]. This marker performed with a sensitivity of 82% for preoperative serum, 93% for peritoneal fluid, and 100% specificity for all tumor types and stages, and therefore, is promising regarding the detection of early ovarian cancer. The analysis of another set of six genes, including *MLH1* and *BRCA1*, with a previously established correlation between the methylation and expression status in borderline versus malignant tumors revealed that promoter methylation was associated with ovarian cancer risk and disease progression [128].

Because sporadic ovarian tumors with hypermethylation of *BRCA1* show a BRCA deficiency phenotype and are, therefore, sensitive to drugs that affect impaired homologous recombination [129], methylation analysis might be a useful tool to predict the suitability of corresponding therapy options, *i.e.*, to individualize therapy protocols [130]. In support of this notion, Stefansson *et al*. [131] showed that *BRCA1* CpG island promoter hypermethylation-associated gene silencing can predict enhanced sensitivity to platinum-derived drugs in cancer cell lines and xenografted tumors, increase the time to relapse, and improve overall survival in ovarian cancer patients undergoing chemotherapy with cisplatin. Finally, the Cancer Genome Atlas Research Network published their integrated genomic analyses of ovarian carcinomas where they performed mRNA and miRNA expression profiling, promoter methylation estimation, DNA copy number detection, and DNA sequencing to analyze the genomes of 489 high-grade serous ovarian carcinomas using various Illumina, Agilent, and Affymetrix platforms [109]. As shown before, *TP53* was frequently mutated in 303 out of 316 samples, and *BRCA1* and *BRCA2* had germline mutations in 8%–9% and somatic mutations in 3% of the cases. *RB1*, *NF1*, *FAT3*, *CSMD3*, *GABRA6*, and *CDK12* were other recurrently mutated genes with statistical significance. Additionally, they found frequent somatic copy number alterations, indicating genomic instability. They proposed that these alterations might be due to the high prevalence of mutations and promoter methylations in DNA repair genes, including homologous recombination components. Strikingly, homologous recombination components were compromised in approximately 50% of the cases, which confirms similar observations by Hilton and colleagues [66]; thus, the authors proposed a benefit of PARP inhibitor therapy for this group [109].

### 4.4. Functional Testing, a Novel Concept in Biomarker Development

A family history of ovarian cancer was convincingly demonstrated to represent the single greatest risk factor [3]. Strikingly similar to breast cancer [25], the vast majority of ovarian cancer susceptibility genes play a role in DNA repair, particularly in the DSB repair pathway of homologous recombination. Moreover, multiple genetic and epigenetic mechanisms cause the inactivation of BRCA1, BRCA2, and/or other repair factors in hereditary and sporadic ovarian cancer, and it was estimated that as much as 50% of epithelial ovarian cancer could be homologous recombination deficient [36,110]. In addition, other genetic factors may influence BRCA function in a fraction of sporadic occurrences. Indeed, families and individuals often harbor more than a single dangerously mutated gene, which requires extended pedigree analysis that in the future may become increasingly difficult in view of the demographic development. Additionally, so-called low-penetrance modifier genes impact high-penetrance risk genes and may confer a significant cancer risk, even in the absence of a susceptibility gene [132].

A common denominator of ovarian cancer risk could be a specific DNA repair deficiency. Analogously to functional assays that detect MSI associated with various mutations in MMR genes in Lynch syndrome patients or that cytogenetically monitor reduced cross-link repair capacities resulting from various FA mutations, it is conceivable that a significant fraction of ovarian cancer risk can be captured by appropriate DNA repair assays (Figure 2). Several groups tested the comet assay and analysis of focal nuclear accumulations (foci) specific for γH2AX or RAD51 to monitor the dysfunction of DSB repair in family members with increased breast and ovarian cancer risk, and in primary ovarian carcinoma cell cultures [36–38]. However, the comet assay and γH2AX analysis quantify DNA breaks and their overall removal, *i.e.*, they do not discriminate between error-free and error-prone mechanisms. The first limitation of RAD51 foci as a marker of HR function is that at least one notable exception among the risk genes exists because BRIP1 is required for HR but dispensable for RAD51 foci formation [133]. Second, hereditary risk is associated with decreased RAD51 foci numbers, *i.e.*, there is a decrease in an already weak signal, often resulting in insufficient sensitivity. However, promising results with an association between inaccurate DNA repair and increased ovarian cancer risk were obtained by assays monitoring micronuclei or distinct error-prone DSB repair pathways in peripheral blood lymphocytes.

#### 4.4.1. Micronucleus Assay

Micronuclei are derived from chromosome fragments arising from asymmetrical structural aberrations or represent whole chromosomes that are not incorporated into the nucleus during cell division. Acentric fragments are most often sequestered within micronuclei after the irradiation of cells, whereas entire chromosomes are more frequently found in spontaneously occurring micronuclei or after spindle poison treatment, as was demonstrated by anti-kinetochore antibody staining [134]. The most frequently used method for the micronucleus test is to score micronuclei in cells that have already passed mitosis and are prevented from cytokinesis by cytochalasin B treatment [135]. In order to define individual DNA repair capacities, blood lymphocytes are isolated, brought into short-term culture, and exposed to ionizing radiation, other genotoxic agents, or left untreated.

Originally, the micronucleus test was used to investigate chromosomal instabilities in patients with mutations in various genes that are needed for the repair of DNA damage, such as in FA [136] or AT patients [137]. The results prompted researchers to look for increased radiosensitivity in the carriers of *BRCA1* or *BRCA2* mutations with the micronucleus test [38]. Indeed, mutation carriers showed, on average, elevated micronucleus frequencies compared with the healthy controls. Baeyens and colleagues [138] further demonstrated that breast cancer patients with *BRCA1* or *BRCA2* mutations showed higher micronucleus frequencies than the controls, but interestingly, there was no difference compared with breast cancer patients without *BRCA1* or *BRCA2* mutations. In this study, healthy *BRCA* mutation carriers showed no difference in the micronucleus test compared with the controls. A meta-analysis by Cardinale *et al*. [139] confirmed a certain degree of data inconsistency. Nevertheless, it could be concluded that, on average, elevated radiation-induced micronucleus frequencies are associated with certain syndromes with compromised DNA repair activities, including *BRCA* mutation carriers, *i.e.*, individuals predisposed to breast and ovarian cancer (Table 1). As mentioned above, ovarian cancer is a heterogeneous disease. Still, the majority of hereditary cases, particularly those with mutations in *BRCA1* and *BRCA2* and, interestingly, a fraction of sporadic ovarian cancer cases with a similar phenotype, have been postulated to share “BRCAness” [18,110]. Taken together, one can speculate that a certain common phenotype is measured by the micronucleus test in irradiated lymphocytes. Thus, despite its limitations, the micronucleus test may represent a valuable functional assay within a range of tests to further characterize this phenotype.

#### 4.4.2. Pathway-Specific DSB Repair Analysis

From the data described above, functional assays turned out to be promising tools for improved assessment of ovarian cancer risk; however, new approaches were needed to specifically address the functional pathways that are most specific for pathogenicity.

In our laboratory, a sensitive test for the detection of specific DNA repair defects was developed more than 10 years ago [140]. This assay system requires two plasmids, which are serially or concomitantly transferred into the cells of interest by either viral infection or DNA transfection. One plasmid carries the information for the expression of the rare-cutting I-*Sce*I restriction enzyme targeting an 18 bp recognition site. The second plasmid contains the DNA substrate for the specific DSB repair pathway of interest. Thus, a typical substrate for homologous DSB repair includes two homologous sequences, a so-called donor and a recipient version of the *EGFP* gene. Both sequences have been mutated such that only after successful DNA recombination, green fluorescing EGFP protein is expressed. The recipient *EGFP* variant carries the I-*Sce*I recognition sequence in place of the chromophore-encoding region, and its expression is driven by the CMV promoter. The donor lacks a promoter element. Specific I-*Sce*I-mediated cleavage within the acceptor sequence creates an artificial DSB. Using the donor sequence as a template, the break is repaired, thus leading to EGFP reactivation. Different *EGFP* donor and recipient sequences have been generated and different donor-recipient combinations cloned, allowing for the specific detection of HR between long and short homologies, SSA, NHEJ, and combinations thereof.

**Figure 3:**
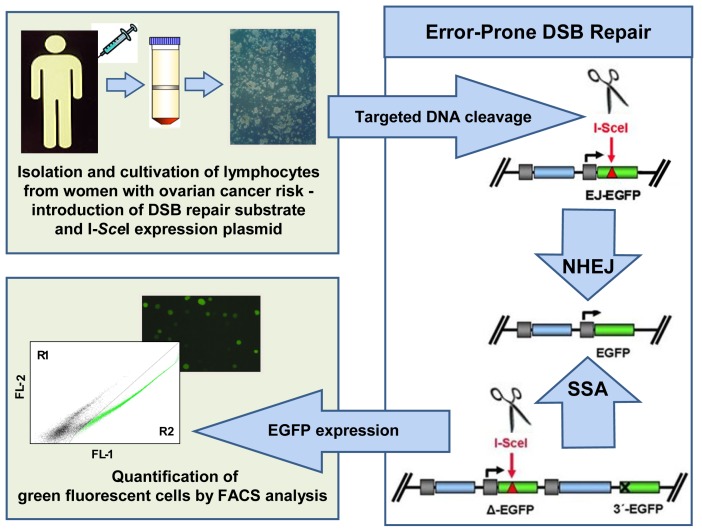
Detection of error-prone DSB repair activities in peripheral blood lymphocytes. Peripheral blood lymphocytes from high-risk breast and ovarian cancer individuals, breast cancer patients, and healthy control individuals were comparatively analyzed using the EGFP-based DSB repair assay system [39]. Peripheral blood lymphocytes were isolated from blood samples by Ficoll gradient centrifugation, brought into *ex vivo* culture, and pathway-specific substrates nucleofected (amaxa) into these lymphocytes together with the expression plasmid for the endonuclease I-*Sce*I. The schematic drawing outlines the representative DNA substrates for measurements of error-prone DSB repair activities, specifically NHEJ and SSA. These substrates are composed of an I-*Sce*I recognition sequence within a mutated EGFP gene, which allows the targeted introduction of a DSB. After NHEJ and SSA, the wild-type *EGFP* gene is reconstituted, and the fraction of EGFP-positive cells is quantified flow cytometrically by FACS analysis.

Taking advantage of this technique, we analyzed a series of lymphoblastoid patient cell lines, *i.e.*, immortalized lymphocytes, with defined mutations in various breast and ovarian cancer predisposing genes including *BRCA1*, *BRCA2*, *TP53*, and *ATM* [140–142]. These studies revealed increases in error-prone DSB repair activities, namely, in microhomology-mediated NHEJ and SSA, as a common denominator. Having identified a phenotypic signature that captures various defects resulting from predisposing genetic alterations, we performed the first case-control study for the prospective evaluation of this potential biomarker in peripheral blood lymphocytes from 35 individuals of breast and ovarian cancer high-risk families, 175 sporadic breast cancer patients, and 245 healthy donors (Figure 3). Primary lymphocytes from blood samples were isolated by Ficoll gradient and cultivated for three days. The DSB repair substrate and I-*Sce*I expression plasmid were introduced into the cells according to amaxa protocols, and then the EGFP-positive cells were quantified by FACS. The results showed that error-prone DSB repair activities were increased in women with familial risk (odds ratios for different DNA substrates: 2.61–4.05) and, to a similar extent, in sporadic breast cancer patients of young age, whereas non-hereditary risk factors had no influence [39]. This effect was observed independently of the presence of *BRCA1/2*-mutations, *i.e.*, it exceeded the limits of genotyping routinely performed in the clinic. Monitoring NHEJ and SSA rather than homologous recombination is more promising for biomarker development because error-prone repair activities increase rather than decrease (as observed with error-free homologous recombination) with hereditary risk, which substantially improves signal sensitivities. The observed shift from homologous recombination to error-prone activities in cells from patients at risk is consistent with earlier models on the accumulation of DNA damage and genomic instabilities in *BRCA1*- and *BRCA2*-mutated cells due to homologous recombination failure [143]. Knowing that hereditary susceptibility is the greatest ovarian cancer risk factor [3] and that all ovarian cancer susceptibility genes except one play a role in DSB repair, these data suggest that the detection of error-prone DSB repair activities in cells derived from blood samples may serve as a powerful tool for ovarian cancer risk assessment.

## 5. Therapy: Reality and Hopes

Ovarian cancer is characterized by vast clinicopathological heterogeneities. Most of the cases show a papillary serous histology (approximately 50%), which is followed by the less common histological variants such as clear cell, endometrioid, and mucinous [144]. Papillary serous and endometrioid tumors, *i.e.*, the majority of tumors, are often presented in advanced stages and tend to be less operable. The histological subtypes also show differences regarding chemoresponsiveness. Whereas papillary serous and endometrioid tumors show responsiveness rates above 60 to 70%, clear cell tumors appear to be much more resistant. Further clinicopathological features, which are important prognostic variables, are stage, histological grade, lymph node involvement, residual tumor size after cytoreductive surgery, ascites, and age. Presently, stage, grade, and residual tumor size show the greatest prognostic values [145].

In light of this review, it is of particular interest that DSB repair dysfunction in familial and sporadic ovarian cancer patients with high-grade serous histology modifies responsiveness to genotoxic chemotherapies, such as the widely used platinum-based regimens [18]. Along the same line, more recent research used synthetic lethality approaches, which revealed the therapeutic efficacy of the better-tolerated PARP inhibitors in DSB repair deficient cancers [143].

### 5.1. Standard of Care and Its Refinement

Even though ovarian cancer encompasses a complex and heterogeneous group of diseases at the clinical, morphological, and molecular levels, therapy strategies do not vary to the same extent. Currently, the key feature for patient outcome is macroscopic complete tumor resection (R0) [146,147]. Several authors reported a favorable prognosis as a consequence of optimal debulking surgery. Although patients can profit from a non-optimal debulking (<1 cm tumor rest R1) as well, macroscopic incomplete resection of the tumor (>2 cm tumor rest R2) is associated with a significantly impaired prognosis in terms of relapse-free and overall survival [148]. The current standard of care is therefore a multidisciplinary approach to primarily achieve R0 resection in ovarian cancer patients. Within the trials investigating the surgery of this disease, the fraction of R0 resection reaches 35 to 45%. Neoadjuvant chemotherapy, therefore, plays a role in the treatment of ovarian cancer, even though it was not possible to demonstrate a survival benefit [149]. The current standard of care in the neoadjuvant chemotherapy setting involves three cycles of chemotherapy before and after surgery [150].

Chemotherapy regimens in the adjuvant setting are platinum based. The standard of care has remained carboplatin in combination with paclitaxel [151]. Several trials demonstrated that carboplatin is as effective as cisplatin but is associated with an improved safety and toxicity profile [151,152]. Other trials investigated alternative carboplatin-based regimens such as carboplatin/PEGylated liposomal doxorubicin or carboplatin/gemcitabine [153,154]. Until now, there has been no evidence of a benefit of a third chemotherapeutic compound to the standard combination. A Japanese group demonstrated a survival benefit with a weekly dose of dense paclitaxel in combination with carboplatin in a phase III trial [155]. Recently, expression data suggested that VEGF could be a target for the treatment of serous and clear cell ovarian carcinomas [94]. Based on positive phase III trial results, bevacizumab, a VEGF antibody, has indeed been approved for the adjuvant therapy of ovarian cancer [156].

In 2003, Cass and colleagues [20] made the surprising observation that a group of ovarian carcinoma patients with *BRCA1* and *BRCA2* mutations had a significantly higher (72%) response rate to primary platinum-based chemotherapy than patients with sporadic disease (36%). The same group also discovered that *in vitro* sensitivity testing to platinum-based chemotherapy was highly predictive of *BRCA* mutation carrier status. Follow-up studies confirmed better response rates of *BRCA* mutation carriers to platinum-based therapy [21,157], highlighting the mechanistic link between platinum-DNA adduct formation and the homologous recombination defect in hereditary ovarian carcinoma patients.

### 5.2. The Potential of PARP Inhibitor Therapy

Tumor cells exhibit a high replication frequency, which increases their dependency on the ability to repair DNA lesions that otherwise will cause DNA replication fork arrest, DSB formation, and ultimately trigger death signals. The detrimental effect of unrepaired DNA lesions can be alleviated by recombinative bypass of stalled replication forks, which requires the integrity of the RAD51-dependent homologous recombination pathway [30,158]. DNA cross-linking agents, including platinum-based chemotherapeutic drugs, are particularly effective at inducing replication fork arrest, which provides a rationale for both cytogenetic FA diagnosis after cross-linker treatment of patient cells [30] and for the observed sensitivity of *BRCA*-mutation carriers to platinum-based chemotherapies [20,157]. However, even in the absence of exogenous genotoxic insults, estrogen-exposed organs such as the ovaries appear to be particularly dependent on an intact homologous recombination pathway, as suggested by the clustering of susceptibility genes in the homologous recombination pathway. This dependency on recombinative replication fork reactivation can possibly be explained by the weak genotoxicity of estrogen metabolites in combination with the proliferation stimulatory effect of the hormones [132].

Because PARP1 is involved in DNA repair at multiple levels [56], it appears to be a good target for the potentiation of the effect of complete loss of BRCA1 or BRCA2 function due to the loss of heterozygosity in hereditary ovarian carcinomas. As PARP-inhibited cells fail to repair DNA damage via base excision repair, single-stranded DNA breaks accumulate and are converted into DSBs when they encounter a DNA replication fork [56,159]. These DSBs lead to fork collapse, which causes cell death without functional homologous recombination. This so-called synthetic lethal interaction between PARP1 and BRCA1 or PARP1 and BRCA2 has caused great excitement [143,160]. Most PARP inhibitors are analogs of the nicotinamide component of NAD^+^ and bind to the catalytic site of PARP, whereby its function is reversible blocked.

In 2005, Bryant *et al*. [161] and Farmer *et al*. [162] were the first to demonstrate that PARP inhibition was lethal for BRCA1- or BRCA2-deficient cells *in vitro* and in tumor xenografts. In 2009, Fong and colleagues [163] published their results from a phase I trial with the PARP inhibitor olaparib involving 60 tumor patients enriched in BRCA mutation carriers, including 16 hereditary ovarian carcinoma patients. This study revealed only very mild toxicities of olaparib and a durable (>4 months), objective antitumor activity (63% benefit: complete response, partial response, stable disease) that occurred only in mutation carriers. These encouraging results prompted further research activities, so that currently, there are 7 PARP inhibitors in clinical trials at different development stages (phases I–III). PARP inhibitors have been studied both as single agents in BRCA-associated or BRCAness cancers and in combination with radiation and/or DNA damaging agents, and also in other tumors [159,164,165].

PARP inhibitors reveal important advantages in comparison with standard cytotoxic chemotherapy, particularly compared with platinum-based therapies. They specifically target homologous recombination-defective tumor cells, *i.e.*, they do not nonspecifically induce DNA damage in all tissues. Therefore, they are relatively nontoxic in normal cells, *i.e.*, they have fewer side effects despite their ability to kill tumor cells. However, as with so many other therapies, resistance to PARP inhibitor treatment has been reported. It has been shown that through second site mutations within the same gene or compensatory mutations within other genes, the HR defect can be reversed [160]. In addition, tumors with increased PARP expression have shown resistance to PARP inhibition. However, the failure of a recent phase III clinical trial with triple-negative breast cancer patients, *i.e.*, sporadic breast cancer patients with frequent defectiveness in the BRCA1 pathway, can simply be explained by the improper use of the drug iniparib, which in fact does not—or, at best, weakly—inhibits PARP [166,167]. In support of the concept that platinum-based chemotherapy and PARP inhibition target the same BRCA-sensitive DNA repair mechanism, replication-associated HR, a correlation between responsiveness towards the two types of treatment was observed in ovarian carcinoma patients [168].

In light of the severe side effects of platinum-based therapies, even when used in combination treatment [153,154], PARP inhibitor treatment remains a promising tool in the therapy of ovarian cancer, at least for the hereditary forms of the disease. Notably, Wang and colleagues [169] showed that the detection of a loss of heterozygosity, another readout for error-prone DNA repair upon homologous recombination failure (Figure 2), enabled the identification of subgroups of high-grade serous carcinomas, which differ with respect to chemotherapy response. A very recent study identified homologous recombination-deficient epithelial ovarian cancers via a RAD51 foci analysis on primary cultures from ascitic fluid. It revealed that homologous recombination deficiency was correlated with higher PARP inhibitor sensitivity *ex vivo*, clinical platinum sensitivity, and improved survival [170]. Future studies are needed to show whether functional assays monitoring homologous recombination failure will also predict the clinical PARP inhibitor response.

## 6. Conclusions

The early detection of ovarian cancer is still lacking powerful biomarker systems, despite extensive research involving imaging technologies, individual serum markers, and array-based omics approaches. Better clues for the identification of increased ovarian cancer risk as a first step towards early detection may come from recent advances in the identification of genetic and functional defects in hereditary cancer patients, which suggest that homologous recombination dysfunction represents the common denominator.

Moreover, with the advent of drugs such as PARP inhibitors that target HR dysfunctional tumors, markers are needed to select those patients who will benefit from treatment. The mutational status of *BRCA1* or *BRCA2* was found to predict therapeutic responsiveness to PARP inhibitor treatment. Future biomarker searches could include BRCA1 modifiers or genes encoding interacting proteins such as *BRIP1*, *BARD1*, or *BAP1* [171]. Functional approaches utilizing patient cells to detect specific DNA repair defects may further complement single gene analysis to evaluate BRCAness, independent of individual mutations, for the development of personalized treatment regarding novel and conventional therapeutics such as PARP inhibitors and carboplatin, respectively.

## Figures and Tables

**Table 1:** Lifetime risks for ovarian cancer susceptibility genes [7–11].

	*BRCA1*	*BRCA2*	*RAD51C*	*RAD51D*
Ovarian cancer	20%–50%	10%–20%	>9%	10%
Breast cancer (female)	50%–90%	40%–60%	* n.s.	n.s.
Breast cancer (male)	0%	11%	0%	?
Prostate cancer	7%	31%	?	?

* n.s., no significant difference.

**Table 2:** Overview of the characteristics of the main epithelial ovarian cancer types [65,67].

Histological subtype of ovarian cancer	Clinicopathological characteristics	Associated gene mutations or pathway defects *
Serous	histology: usually large; often bilateral; mixture of cystic, papillary, and solid growth; usually marked nuclear atypia	
	low-grade: slow proliferation/progression; poor response to chemotherapy	low-grade: *BRAF* and *KRAS* mutations (Ras/Raf/MEK/MAPK pathway)
	high-grade: rapid proliferation/progression; poor response to chemotherapy; recurrence	high-grade: germline *BRCA1*/*2* mutations; *TP53* mutations; *HER2*/*neu* amplifications; *AKT2* amplifications (PI3K pathway)
Endometrioid	histology: strong resemblance to endometrial adenocarcinoma; associated with endometriosis	*PTEN* mutations (PI3K pathway); *β-catenin* mutations (WNT signaling); Microsatellite instability via loss of MMR gene expression; high-grade: *TP53* mutations
Clear cell	histology: growth tubular, papillary, solid, or frequently mixed types; associated with endometriosis	PI3K pathway deregulation; Microsatellite instability in subset; low frequency of *KRAS*, *BRAF*, and *TP53* mutations
Mucinous	histology: usually unilocular; ~largest of all ovarian tumors; papillary and solid forms; associated with endometriosis; poor response to chemotherapy compared with serous cancer	frequently KRAS mutations; overexpression of mucin genes (*MUC2*, *MUC3*, and *MUC17*)

* Associated gene mutations or pathway defects that play critical roles in the etiology are described in this table. The table is not intended to be exhaustive because the transition between certain types is fluid.

## Abbreviations

AT: Ataxia telangiectasia
ATM: Ataxia telangiectasia mutated
Bcl-2: B-cell lymphoma 2
BARD1: BRCA1-associated RING domain protein 1
BACH1: BRCA1-associated *C*-terminal helicase 1
BAP1: BRCA1-associated protein 1
BRCA1: breast-cancer susceptibility gene 1
BRCA2: breast-cancer susceptibility gene 2
BRIP1: BRCA1 interacting protein 1
CA125: cancer antigen 125
Chk2: checkpoint kinase 2
CAN: acquired somatic copy number aberration
CNV: copy number variation
CAT: computerized axial tomography
CtIP: *C*-terminal-binding protein interacting protein
DSB: DNA double-strand break
EGFP: enhanced green fluorescent protein
EGFR: epidermal growth factor receptor
FACS: fluorescence activated cell sorting
FA: Fanconi anemia
HER2: human epidermal growth factor receptor 2
HE4: human epididymis protein 4
FIGO: International Federation of Obstetricians and Gynecologists
LFS: Li-Fraumeni syndrome
MAPK: mitogen-activated protein kinase
MEK: MAPK/ERK kinase
MGMT: O6-methylguanine-DNA-methyltransferase
MMR: mismatch repair
MLPA: multiplex ligation-dependent probe amplification
MRE11: meiotic recombination 11
MRN-complex: protein complex consisting of MRE11, RAD50, and Nibrin
MLH: MutL homolog
MSH: MutS homolog
NHEJ: non-homologous end-joining
MRI: nuclear magnetic resonance imaging
NER: nucleotide excision repair
PALB2: partner and localizer of BRCA2
PJS: Peutz-Jeghers syndrome
PI3K: phosphatidylinositol 3-kinase
PTEN: phosphatase and tensin homolog
γH2AX: phospho-histone AX
PARP: Poly(ADP-ribose)polymerase
PMS: postmeiotic segregation
RAD51C: RAD51 homolog C
RAD51D: RAD51 homolog D
Ras: rat sarcoma
Raf: rapidly accelerated fibrosarcoma or rat fibrosarcoma
ROMA: risk of ovarian malignancy algorithm
STK11: serine/threonine kinase 11
SNP: single nucleotide polymorphism
SSA: single-strand annealing
TNFα: tumor necrosis factor α
*TP53*: gene encoding human p53

## References

[1] Hunn J., Rodriguez G.C. (2012). Ovarian cancer: Etiology, risk factors, and epidemiology. Clin. Obstet. Gynecol.

[2] Altekruse S.F., Kosary C.L., Krapcho M., Neuman N., Aminou R., Waldron W., Ruhl J., Howlader N., Tatalovich Z., Cho H. SEER Cancer Statistics Review, 1975–2009.

[3] Stratton J.F., Pharoah P., Smith S.K., Easton D., Ponder B.A. (1998). A systematic review and meta-analysis of family history and risk of ovarian cancer. Br. J. Obstet. Gynaecol.

[4] Miki Y., Swensen J., Shattuck-Eidens D., Futreal P.A., Harshman K., Tavtigian S., Liu Q., Cochran C., Bennett L.M., Ding W. (1994). A strong candidate for the breast and ovarian cancer susceptibility gene BRCA1. Science.

[5] Wooster R., Bignell G., Lancaster J., Swift S., Seal S., Mangion J., Collins N., Gregory S., Gumbs C., Micklem G. (1995). Identification of the breast cancer susceptibility gene BRCA2. Nature.

[6] Meindl A., Hellebrand H., Wiek C., Erven V., Wappenschmidt B., Niederacher D., Freund M., Lichtner P., Hartmann L., Schaal H. (2010). Germline mutations in breast and ovarian cancer pedigrees establish RAD51C as a human cancer susceptibility gene. Nat. Genet.

[7] Loveday C., Turnbull C., Ruark E., Xicola R.M., Ramsay E., Hughes D., Warren-Perry M., Snape K., Eccles D., Breast Cancer Susceptibility Collaboration (UK) (2012). Germline RAD51C mutations confer susceptibility to ovarian cancer. Nat. Genet.

[8] Antoniou A., Pharoah P.D., Narod S., Risch H.A., Eyfjord J.E., Hopper J.L., Loman N., Olsson H., Johannsson O., Borg A. (2003). Average risks of breast and ovarian cancer associated with BRCA1 or BRCA2 mutations detected in case series unselected for family history: A combined analysis of 22 studies. Am. J. Hum. Genet.

[9] Risch H.A., McLaughlin J.R., Cole D.E., Rosen B., Bradley L., Fan I., Tang J., Li S., Zhang S., Shaw P.A. (2006). Population BRCA1 and BRCA2 mutation frequencies and cancer penetrances: A kin-cohort study in Ontario, Canada. J. Natl. Cancer Inst.

[10] Chen S., Parmigiani G. (2007). Meta-analysis of BRCA1 and BRCA2 penetrance. J. Clin. Oncol.

[11] Loveday C., Turnbull C., Ramsay E., Hughes D., Ruark E., Frankum J.R., Bowden G., Kalmyrzaev B., Warren-Perry M., Snape K. (2011). Germline mutations in RAD51D confer susceptibility to ovarian cancer. Nat. Genet.

[12] Lynch H.T., Casey M.J., Snyder C.L., Bewtra C., Lynch J.F., Butts M., Godwin A.K. (2009). Hereditary ovarian cancer: Molecular genetics, pathology, management, and heterogeneity. Mol. Oncol.

[13] Pennington K.P., Swisher E.M. (2012). Hereditary ovarian cancer: Beyond he usual suspects. Gynecol. Oncol.

[14] Sourbier C. (2012). Ovarian cancer: Emerging molecular-targeted therapies. Biologics.

[15] Slomski A. (2012). Screening women for ovarian cancer still does more harm than good. JAMA.

[16] Longuespee R., Boyon C., Desmons A., Vinatier D., Leblanc E., Farre I., Wisztorski M., Ly K., D’Anjou F., Day R. (2012). Ovarian cancer molecular pathology. Cancer Metastasis Rev.

[17] Valentini A.L., Gui B., Miccò M., Mingote M.C., De Gaetano A.M., Ninivaggi V., Bonomo L. (2012). Benign and suspicious ovarian masses—MR imaging criteria for characterization: Pictorial review. J. Oncol.

[18] Rigakos G., Razis E. (2012). BRCAness: Finding the achilles heel in ovarian cancer. Oncologist.

[19] Claus E.B., Schwartz P.E. (1995). Familial ovarian cancer. Update and clinical applications. Cancer.

[20] Cass I., Baldwin R.L., Varkey T., Moslehi R., Narod S.A., Karlan B.Y. (2003). Improved survival in women with *BRCA*-associated ovarian carcinoma. Cancer.

[21] Alsop K., Fereday S., Meldrum C., deFazio A., Emmanuel C., George J., Dobrovic A., Birrer M.J., Webb P.M., Stewart C. (2012). BRCA mutation frequency and patterns of treatment response in BRCA mutation-positive women with ovarian cancer: A report from the Australian Ovarian Cancer Study Group. J. Clin. Oncol.

[22] Kinzler K.W., Vogelstein B. (1997). Cancer-susceptibility genes. Gatekeepers and caretakers. Nature.

[23] Howlett N.G., Taniguchi T., Durkin S.G., D’Andrea A.D., Glover T.W. (2005). The Fanconi anemia pathway is required for the DNA replication stress response and for the regulation of common fragile site stability. Hum. Mol. Genet.

[24] Kennedy R.D., D’Andrea A.D. (2005). The Fanconi anemia/BRCA pathway: New faces in the crowd. Genes Dev.

[25] Walsh T., King M.C. (2007). Ten genes for inherited breast cancer. Cancer Cell.

[26] Surtees J.A., Argueso J.L., Alani E. (2004). Mismatch repair proteins: Key regulators of genetic recombination. Cytogenet. Genome Res.

[27] Siehler S.Y., Schrauder M., Gerischer U., Cantor S., Marra G., Wiesmüller L. (2009). Human Mlh1 monitors homologous recombination independently of mismatch repair and damage signaling. DNA Repair.

[28] Lavin M.F. (2008). Ataxia-telangiectasia: From a rare disorder to a paradigm for cell signalling and cancer. Nat. Rev. Mol. Cell Biol.

[29] Gatz S.A., Wiesmüller L. (2006). p53 in recombination and repair. Cell Death Differ.

[30] Thompson L.H., Hinz J.M. (2009). Cellular and molecular consequences of defective Fanconi anemia proteins in replication-coupled DNA repair: Mechanistic insights. Mutat. Res.

[31] O’Brien V., Brown R. (2006). Signalling cell cycle arrest and cell death through the MMR system. Carcinogenesis.

[32] Ewald B., Sampath D., Plunkett W. (2008). ATM and the Mre11-Rad50-Nbs1 complex respond to nucleoside analogue-induced stalled replication forks and contribute to drug resistance. Cancer Res.

[33] Sato K., Ohta T., Venkitaraman A.R. (2010). A mitotic role for the DNA damage-responsive CHK2 kinase. Nat. Cell Biol.

[34] Hombauer H., Srivatsan A., Putnam C.D., Kolodner R.D. (2011). Mismatch repair, but not heteroduplex rejection, is temporally coupled to DNA replication. Science.

[35] Schlacher K., Wu H., Jasin M. (2012). A distinct replication fork protection pathway connects Fanconi anemia tumor suppressors to RAD51-BRCA1/2. Cancer Cell.

[36] Mukhopadhyay A., Elattar A., Cerbinskaite A., Wilkinson S.J., Drew Y., Kyle S., Los G., Hostomsky Z., Edmondson R.J., Curtin N.J. (2010). Development of a functional assay for homologous recombination status in primary cultures of epithelial ovarian tumor and correlation with sensitivity to poly(ADP-ribose) polymerase inhibitors. Clin. Cancer Res..

[37] Rothfuss A., Schütz P., Bochum S., Volm T., Eberhardt E., Kreienberg R., Vogel W., Speit G. (2000). Induced micronucleus frequencies in peripheral lymphocytes as a screening test for carriers of a BRCA1 mutation in breast cancer families. Cancer Res.

[38] Redon C.E., Nakamura A.J., Zhang Y.W., Ji J.J., Bonner W.M., Kinders R.J., Parchment R.E., Doroshow J.H., Pommier Y. (2010). Histone γH2AX and Poly(ADP-Ribose) as clinical pharmacodynamic biomarkers. Clin. Cancer Res.

[39] Keimling M., Deniz M., Varga D., Stahl A., Schrezenmeier H., Kreienberg R., Hoffmann I., König J., Wiesmüller L. (2012). The power of DNA double-strand break (DSB) repair testing to predict breast cancer susceptibility. FASEB J.

[40] Roy R., Chun J., Powell S.N. (2011). BRCA1 and BRCA2: Different roles in a common pathway of genome protection. Nat. Rev. Cancer.

[41] Somyajit K., Subramanya S., Nagaraju G. (2010). RAD51C: A novel cancer susceptibility gene is linked to FANconi anemia and breast cancer. Carcinogenesis.

[42] Bozzao C., Lastella P., Stella A. (2011). Anticipation in lynch syndrome: Where we are where we go. Curr. Genomics.

[43] Thorstenson Y.R., Roxas A., Kroiss R., Jenkins M.A., Yu K.M., Bachrich T., Muhr D., Wayne T.L., Chu G., Davis R.W. (2003). Contributions of ATM mutations to familial breast and ovarian cancer. Cancer Res.

[44] Roemer K. (1999). Mutant p53: Gain-of-function oncoproteins and wild-type p53 inactivators. Biol. Chem.

[45] Malkin D. (2011). Li-Fraumeni syndrome. Genes Cancer.

[46] McCuaig J.M., Armel S.R., Novokmet A., Ginsburg O.M., Demsky R., Narod S.A., Malkin D. (2012). Routine TP53 testing for breast cancer under age 30: Ready for prime time?. Fam. Cancer.

[47] Bertrand P., Saintigny Y., Lopez B.S. (2004). p53’s double life: Transactivation-independent repression of homologous recombination. Trends Genet.

[48] Shen W.H., Balajee A.S., Wang J., Wu H., Eng C., Pandolfi P.P., Yin Y. (2007). Essential role for nuclear PTEN in maintaining chromosomal integrity. Cell.

[49] Jin S., Mao H., Schnepp R.W., Sykes S.M., Silva A.C., D’Andrea A.D., Hua X. (2003). Menin associates with FANCD2, a protein involved in repair of DNA damage. Cancer Res.

[50] Marek L.R., Kottemann M.C., Glazer P.M., Bale A.E. (2008). MEN1 and FANCD2 mediate distinct mechanisms of DNA crosslink repair. DNA Repair.

[51] Gallo A., Agnese S., Esposito I., Galgani M., Avvedimento V.E. (2010). Menin stimulates homology-directed DNA repair. FEBS Lett.

[52] Lindor N.M., McMaster M.L., Lindor C.J., Greene M.H. (2008). Concise handbook of familial cancer susceptibility syndromes- second edition. J. Natl. Cancer Inst. Monogr.

[53] Hoeijmakers J.H. (2001). Genome maintenance mechanisms for preventing cancer. Nature.

[54] Jackson S.P., Bartek J. (2009). The DNA-damage response in human biology and disease. Nature.

[55] Schrauder M., Wiesmüller L., Debatin K.M., Fulda S. (2006). DNA Repair. Apoptosis and Cancer Therapy.

[56] Caldecott K.W. (2008). Single-strand break repair and genetic disease. Nat. Rev. Genet.

[57] Liu Y., Kadyrov F.A., Modrich P. (2011). PARP-1 enhances the mismatch-dependence of 5′-directed excision in human mismatch repair in vitro. DNA Repair.

[58] Huertas P. (2010). DNA resection in eukaryotes: Deciding how to fix the break. Nat. Struct. Mol. Biol.

[59] Weterings E., Chen D.J. (2008). The endless tale of non-homologous end-joining. Cell Res.

[60] O’Donovan P.J., Livingston D.M. (2010). BRCA1 and BRCA2: Breast/ovarian cancer susceptibility gene products and participants in DNA double-strand break repair. Carcinogenesis.

[61] Nussenzweig A., Nussenzweig M.C. (2007). A backup DNA repair pathway moves to the forefront. Cell.

[62] Bryant H.E., Petermann E., Schultz N., Jemth A.S., Loseva O., Issaeva N., Johansson F., Fernandez S., McGlynn P., Helleday T. (2009). PARP is activated at stalled forks to mediate Mre11-dependent replication restart and recombination. EMBO J.

[63] Malpica A., Deavers M.T., Lu K., Bodurka D.C., Atkinson E.N., Gershenson D.M., Silva E.G. (2004). Grading ovarian serous carcinoma using a two-tier system. Am. J. Surg. Pathol.

[64] Shih I., Kurman R.J. (2004). Ovarian tumorigenesis: A proposed model based on morphological and molecular genetic analysis. Am. J. Pathol.

[65] Cho K.R., Shih I. (2009). Ovarian cancer. Annu. Rev. Pathol.

[66] Merritt M.A., Cramer D.W. (2010). Molecular pathogenesis of endometrial and ovarian cancer. Cancer Biomarkers.

[67] Kaku T., Ogawa S., Kawano Y., Ohishi Y., Kobayashi H., Hirakawa T., Nakano H. (2003). Histological classification of ovarian cancer. Med. Electron. Microsc.

[68] Cai K.Q., Albarracin C., Rosen D., Zhong R., Zheng W., Luthra R., Broaddus R., Liu J. (2004). Microsatellite instability and alteration of the expression of hMLH1 and hMSH2 in ovarian clear cell carcinoma. Hum. Pathol.

[69] Naik J.D., Seligmann J., Perren T.J. (2012). Mucinous tumours of the ovary. J. Clin. Pathol.

[70] Wamunyokoli F.W., Bonome T., Lee J.Y., Feltimate C.M., Welch W.R., Radonovich M., Pise-Masison C., Brady J., Hao K., Berkowitz R.S. (2006). Expression profiling of mucinous tumors of the ovary identifies genes of clinicopathologic importance. Clin. Cancer Res.

[71] Koh S.C., Razvi K., Chan Y.H., Narasimhan K., Ilancheran A., Low J.J., Choolani M., Ovarian Cancer Research Consortium of SE Asia (2011). The association with age, human tissue kallikreins 6 and 10 and hemostatic markers for survival outcome from epithelial ovarian cancer. Arch. Gynecol. Obstet..

[72] Meden H., Fattahi-Meibodi A. (1998). CA 125 in benign gynecological conditions. Int. J. Biol. Markers.

[73] Kim J.H., Skates S.J., Uede T., Wong K.K., Schorge J.O., Feltmate C.M., Berkowitz R.S., Cramer D.W., Mok S.C. (2002). Osteopontin as a potential diagnostic biomarker for ovarian cancer. JAMA.

[74] Diamandis E.P., Scorilas A., Fracchioli S., van Gramberen M., de Bruijn H., Henrik A., Soosaipillai A., Grass L., Yousef G.M., Stenman U.H. (2003). Human kallikrein 6 (hK6): A new potential serum biomarker for diagnosis and prognosis of ovarian carcinoma. J. Clin. Oncol.

[75] Kishi T., Grass L., Soosaipillai A., Scorilas A., Harbeck N., Schmalfeldt B., Dorn J., Mysliwiec M., Schmitt M., Diamandis E.P. (2003). Human kallikrein 8, a novel biomarker for ovarian carcinoma. Cancer Res.

[76] Matsuzaki H., Kobayashi H., Yagyu T., Wakahara K., Kondo T., Kurita N., Sekino H., Inagaki K., Suzuki M., Kanayama N. (2005). Plasma bikunin as a favorable prognostic factor in ovarian cancer. J. Clin. Oncol.

[77] Nowee M.E., Snijders A.M., Rockx D.A., de Wit R.M., Kosma V.M., Hämäläinen K., Schouten J.P., Verheijen R.H., van Diest P.J., Albertson D.G. (2007). DNA profiling of primary serous ovarian and fallopian tube carcinomas with array comparative genomic hybridization and multiplex ligation-dependent probe amplification. J. Pathol.

[78] Bandiera E., Franceschini R., Specchia C., Bignotti E., Trevisiol C., Gion M., Pecorelli S., Santin A.D., Ravaggi A. (2012). Prognostic significance of vascular endothelial growth factor serum determination in women with ovarian cancer. ISRN Obstet. Gynecol..

[79] Moore R.G., Brown A.K., Miller M.C., Skates S., Allard W.J., Verch T., Steinhoff M., Messerlian G., DiSilvestro P., Granai C.O. (2008). The use of multiple novel tumor biomarkers for the detection of ovarian carcinoma in patients with a pelvic mass. Gynecol. Oncol.

[80] Yurkovetsky Z., Skates S., Lomakin A., Nolen B., Pulsipher T., Modugno F., Marks J., Godwin A., Gorelik E., Jacobs I. (2010). Development of a multimarker assay for early detection of ovarian cancer. J. Clin. Oncol.

[81] Chan K.K., Chen C.A., Nam J.H., Ochiai K., Wilailak S., Choon A.T., Sabaratnam S., Hebbar S., Sickan J., Schodin B.A. (2012). The use of HE4 in the prediction of ovarian cancer in Asian women with a pelvic mass. Gynecol. Oncol..

[82] Escudero J.M., Auge J.M., Filella X., Torne A., Pahisa J., Molina R. (2011). Comparison of serum human epididymis protein 4 with cancer antigen 125 as a tumor marker in patients with malignant and nonmalignant diseases. Clin. Chem..

[83] Freydanck M.K., Laubender R.P., Rack B., Schumacher L., Jeschke U., Scholz C. (2012). Two-marker combinations for preoperative discrimination of benign and malignant ovarian masses. Anticancer Res.

[84] Van Gorp T., Cadron I., Despierre E., Daemen E., Daemen A., Leunen K., Amant F., Timmermann D., de Moor B., Vergote I. (2011). HE4 and CA125 as a diagnostic test in ovarian cancer: Prospective validation oft he risk of ovarian malignancy algorithm. Br. J. Cancer.

[85] Van Gorp T., Veldman J., van Calster B., Cadron I., Leunen K., Amant F., Timmermann D., Vergote I. (2012). Subjective assessment by ultrasound is superior to the risk of malignancy index (RMI) or the risk of ovarian malignancy algorithm (ROMA) in discriminating benign from malignant adnexal masses. Eur. J. Cancer.

[86] Li F., Tie R., Chang K., Wang F., Deng S., Lu W., Yu L., Chen M. (2012). Does risk for ovarian malignancy algorithm excel human epididymis protein 4 and ca125 in predicting epithelial ovarian cancer. A meta-analysis. BMC Cancer.

[87] Trudel D., Tetu B., Gregoire J., Plante M., Renaud M.C., Bachvarov D., Douville P., Bairate I. (2012). Human epididymis protein 4 (HE4) and ovarian cancer prognosis. Gynecol. Oncol..

[88] Kong S.Y., Han M.H., Yoo H.J., Hwang J.H., Lim M.C., Seo S.S., Yoo C.W., Kim J.H., Park S.Y., Kang S. (2012). Serum HE4 level is an independent prognostic factor in epithelial ovarian cancer. Ann. Surg. Oncol.

[89] Georgakopoulos P., Mehmood S., Akalin A., Shroyer K.R. (2012). Immunohistochemical localization of HE4 in benign, borderline, and malignant lesions of the ovary. Int. J. Gynecol. Pathol.

[90] Novotny Z., Presl J., Kucera R., Topolcan O., Vrzalova J., Fuchsova R., Betincova L., Rokyta Z. (2012). HE4 and ROMA index in Czech postmenopausal women. Anticancer Res.

[91] Urban N., Thorpe J., Karlan B.Y., McIntosh M.W., Palomares M.R., Daly M.B., Paley P., Drescher C.W. (2012). Interpretation of single and serial measures of HE4 and CA125 in asymptomatic women at high risk for ovarian cancer. Cancer Epidemiol. Biomarkers Prev.

[92] Suh K.S., Park S.W., Castro A., Patel H., Blake M.L., Goy A. (2010). Ovarian cancer biomarkers for molecular biosensors and translational medicine. Expert Rev. Mol. Diagn..

[93] Zhang B., Barekati Z., Kohler C., Radpour R., Asadollahi R., Holzgreve W., Zhong X.Y. (2010). Proteomics and biomarkers for ovarian cancer diagnosis. Ann. Clin. Lab. Sci.

[94] Nolen B.M., Lokshin A.E. (2012). Protein biomarkers of ovarian cancer: The forest and the trees. Future Oncol.

[95] Le Page C., Huntsman D.G., Provencher D.M., Mes-Masson A.-M. (2010). Predictive and prognostic protein biomarkers in epithelial ovarian cancer: Recommendation for future studies. Cancers.

[96] Visintin I., Feng Z., Longton G., Ward D.C., Alvero A.B., Lai Y., Tenthorey J., Leiser A., Flores-Saaib R., Yu H. (2008). Diagnostic markers for early detection of ovarian cancer. Clin. Cancer Res.

[97] Mcintosh M., Anderson G., Drescher C., Hanash S., Urban N., Brown P., Gambhir S.S., Coukos G., Laird P.W., Nelson B. (2008). Ovarian cancer early detection claims are biased. Clin. Cancer Res.

[98] Zhang Z., Bast R.C., Yu Y., Li J., Sokoll L.J., Rai A.J., Rosenzweig J.M., Cameron B., Wang Y.Y., Meng X.Y. (2004). Three biomarkers identified from serum proteomic analysis for the detection of early stage ovarian cancer. Cancer Res..

[99] Skates S.J., Horick N., Yu Y., Xu F.J., Berchuck A., Havrilesky L.J., de Bruijn H.W., van der Zee A.G., Woolas R.P., Jacobs I.J. (2004). Preoperative sensitivity and specificity for early-stage ovarian cancer when combining cancer antigen CA-125II, CA 15–3, CA 72–4, and macrophage colony-stimulating factor using mixtures of multivariate normal distributions. J. Clin. Oncol.

[100] Amonkar S.D., Bertenshaw G.P., Chen T.H., Bergstrom K.J., Zhao J., Seshaiah P., Yip P., Mansfield B.C. (2009). Development and preliminary evaluation of a multivariate index assay for ovarian cancer. PLoS One.

[101] Feuk L., Carson A.R., Scherer S.W. (2006). Structural variation in the human genome. Nat. Rev. Genet.

[102] Alkan C., Bradley P.C., Eichler E. (2011). Genome structural variation discovery and genotyping. Nat. Rev.

[103] Stankiewicz P., Lupski J.R. (2010). Structural variation in the human genome and its role in disease. Annu. Rev. Med.

[104] Stranger B.E., Forrest M.S., Dunning M., Ingle C.E., Beazley C., Thorne N., Redon R., Bird C.P., de Grassi A., Lee C. (2007). Relative impact of nucleotide and copy number variation on gene expression phenotypes. Science.

[105] Albertson D.G., Collins C., McCormick F., Gray J.W. (2003). Chromosome aberrations in solid tumors. Nat. Genet.

[106] Kalb R., Neveling K., Nanda I., Schindler D., Hoehn H., Volff J.N. (2006). Fanconi Anemia Causes and Consequences of Genetic Instability. Genome and Disease. Genome Dyn.

[107] Negrini S., Gorgoulis V.G., Halazonetis T.D. (2010). Genomic instability—An evolving hallmark of cancer. Nat. Rev. Mol. Cell Biol.

[108] Engert S., Wappenschmidt B., Betz B., Kast K., Kutsche M., Hellebrand H., Goecke T.O., Kiechle M., Niederacher D., Schmutzler R.K. (2008). MLPA screening in the BRCA1 gene from 1506 German hereditary breast cancer cases: Novel deletions, frequent involvement of exon 17, and occurrence in single early-onset cases. Hum. Mutat.

[109] The Cancer Genome Atlas Research Network (2011). Integrated genomic analyses of ovarian carcinoma. Nature.

[110] Hilton J.L., Geisler J.P., Rathe J.A., Hattermann-Zogg M.A., DeYoung B., Buller R.E. (2002). Inactivation of BRCA1 and BRCA2 in ovarian cancer. J. Natl. Cancer Inst.

[111] Nakayama K., Nakayama N., Kurman R.J., Cope L., Pohl G., Samuels Y., Velculescu V.E., Wang T.L., Shih I.M. (2006). Sequence mutations and amplifications of PIK3CA and AKT2 genes in purified ovarian serous neoplasms. Cancer Biol. Ther.

[112] Engler D.A., Gupta S., Growdon W.B., Drapkin R.I., Nitta M., Sergent P.A., Allred S.F., Gross J., Deavers M.T., Kuo W.L. (2012). Genome wide DNA copy number analysis of serous type ovarian carcinomas identifies genetic markers predictive of clinical outcome. PLoS One.

[113] Gorringe K.L., George J., Anglesio M.S., Ramakrishna M., Etemadmoghadam D., Cowin P., Sridhar A., Williams L.H., Boyle S.E., Yanaihara N. (2010). Copy number analysis identifies novel interactions between genomic loci in ovarian cancer. PLoS One.

[114] Schwartz D.R., Kardia S.L., Shedden K.A., Kuick R., Michailidis G., Taylor J.M., Misek D.E., Wu R., Zhai Y., Darrah D.M. (2002). Gene expression in ovarian cancer reflects both morphology and biological behavior, distinguishing clear cell from other poor-prognosis ovarian carcinomas. Cancer Res.

[115] Bonome T., Lee J.Y., Park D.C., Radonovich M., Pise-Masison C., Brady J., Gardner G.J., Hao K., Wong W.H., Barrett J.C. (2005). Expression profiling of serous low malignant potential, low-grade, and high-grade tumors of the ovary. Cancer Res.

[116] Tothill R.W., Tinker A.V., George J., Brown R., Fox S.B., Lade S., Johnson D.S., Trivett M.K., Etemadmoghadam D., Locandro B. (2008). Novel molecular subtypes of serous and endometrioid ovarian cancer linked to clinical outcome. Clin. Cancer Res.

[117] Jazaeri A.A., Awtrey C.S., Chandramouli G.V., Chuang Y.E., Khan J., Sotiriou C., Aprelikova O., Yee C.J., Zorn K.K., Birrer M.J. (2005). Gene expression profiles associated with response to chemotherapy in epithelial ovarian cancers. Clin. Cancer Res.

[118] Fekete T., Rásó E., Pete I., Tegze B., Liko I., Munkácsy G., Sipos N., Rigó J., Györffy B. (2012). Meta-analysis of gene expression profiles associated with histological classification and survival in 829 ovarian cancer samples. Int. J. Cancer.

[119] Kang J., D’Andrea A.D., Kozono D. (2012). A DNA repair pathway-focused score for prediction of outcomes in ovarian cancer treated with platinum-based chemotherapy. J. Natl. Cancer Inst.

[120] Zeller C., Dai W., Steele N.L., Siddiq A., Walley A.J., Wilhelm-Benartzi C.S., Rizzo S., van der Zee A., Plumb J.A., Brown R. (2012). Candidate DNA methylation drivers of acquired cisplatin resistance in ovarian cancer identified by methylome and expression profiling. Oncogene.

[121] Balch C., Matei D.E., Huang T.H., Nephew K.P. (2010). Role of epigenomics in ovarian and endometrial cancers. Epigenomics.

[122] Jones P.A., Baylin S.B. (2007). The epigenomics of cancer. Cell.

[123] Baldwin R.L., Nemeth E., Tran H., Shvartsman H., Cass I., Narod S., Karlan B.Y. (2000). BRCA1 promoter region hypermethylation in ovarian carcinoma: A population-based study. Cancer Res.

[124] Strathdee G., Appleton K., Illand M., Millan D.W., Sargent J., Paul J., Brown R. (2001). Primary ovarian carcinomas display multiple methylator phenotypes involving known tumor suppressor genes. Am. J. Pathol.

[125] Rathi A., Virmani A.K., Schorge J.O., Elias K.J., Maruyama R., Minna J.D., Mok S.C., Girard L., Fishman D.A., Gazdar A.F. (2002). Methylation profiles of sporadic ovarian tumors and nonmalignant ovaries from high-risk women. Clin. Cancer Res.

[126] Teodoridis J.M., Hall J., Marsh S., Kannall H.D., Smyth C., Curto J., Siddiqui N., Gabra H., McLeod H.L., Strathdee G. (2005). CpG island methylation of DNA damage response genes in advanced ovarian cancer. Cancer Res.

[127] De Caceres I., Battagli C., Esteller M., Herman J.G., Dulaimi E., Edelson M.I., Bergman C., Ehya H., Eisenberg B.L., Cairns P. (2004). Tumor cell-specific BRCA1 and RASSF1A hypermethylation in serum, plasma, and peritoneal fluid from ovarian cancer patients. Cancer Res.

[128] Wiley A., Katsaros D., Chen H., Rigault de la Longrais I.A., Beeghly A., Puopolo M., Singal R., Zhang Y., Amoako A., Zelterman D. (2006). Aberrant promoter methylation of multiple genes in malignant ovarian tumors and in ovarian tumors with low malignant potential. Cancer.

[129] Turner N., Tutt A., Ashworth A. (2004). Hallmarks of “BRCAness” in sporadic cancers. Nat. Rev. Cancer.

[130] Maradeo M.E., Cairns P. (2011). Translational application of epigenetic alterations: Ovarian cancer as a model. FEBS Lett.

[131] Stefansson O.A., Villanueva A., Vidal A., Martí L., Esteller M. (2012). BRCA1 epigenetic inactivation predicts sensitivity to platinum-based chemotherapy in breast and ovarian cancer. Epigenetics.

[132] Ralhan R., Kaur J., Kreienberg R., Wiesmüller L. (2007). Links between DNA double strand break repair and breast cancer: Accumulating evidence from both familial and nonfamilial cases. Cancer Lett.

[133] Litman R., Peng M., Jin Z., Zhang F., Zhang J., Powell S., Andreassen P.R., Cantor S.B. (2005). BACH1 is critical for homologous recombination and appears to be the Fanconi anemia gene product FANCJ. Cancer Cell.

[134] Fenech M., Morley A.A. (1989). Kinetochore detection in micronuclei: An alternative method for measuring chromosome loss. Mutagenesis.

[135] Fenech M. (2000). The *in vitro* micronucleus technique. Mutat. Res.

[136] Zunino A., Degan P., Vigo T., Abbondandolo A. (2001). Hydrogen peroxide: Effects on DNA, chromosomes, cell cycle and apoptosis induction in Fanconi’s anemia cell lines. Mutagenesis.

[137] Gutiérrez-Enríquez S., Hall J. (2003). Use of the cytokinesis-block micronucleus assay to measure radiation-induced chromosome damage in lymphoblastoid cell lines. Mutat. Res.

[138] Baeyens A., Thierens H., Claes K., Poppe B., de Ridder L., Vral A. (2004). Chromosomal radiosensitivity in BRCA1 and BRCA2 mutation carriers. Int. J. Radiat. Biol.

[139] Cardinale F., Bruzzi P., Bolognesi C. (2012). Role of micronucleus test in predicting breast cancer susceptibility: A systematic review and meta-analysis. Br. J. Cancer.

[140] Akyüz N., Boehden G.S., Süsse S., Rimek A., Preuss U., Scheidtmann K.H., Wiesmüller L. (2002). DNA substrate dependence of p53-mediated regulation of double-strand break repair. Mol. Cell Biol.

[141] Keimling M., Kaur J., Bagadi S.A., Kreienberg R., Wiesmüller L., Ralhan R. (2008). A sensitive test for the detection of specific DSB repair defects in primary cells from breast cancer specimens. Int. J. Cancer.

[142] Keimling M., Volcic M., Csernok A., Wieland B., Dörk T., Wiesmüller L. (2011). Functional characterization connects individual patient mutations in ataxia telangiectasia mutated (ATM) with dysfunction of specific DNA double-strand break-repair signaling pathways. FASEB J.

[143] Ashworth A. (2008). A synthetic lethal therapeutic approach: Poly(ADP) ribose polymerase inhibitors for the treatment of cancers deficient in DNA double-strand break repair. J. Clin. Oncol.

[144] Romero I., Bast R.C. (2012). Minireview: Human ovarian cancer: Biology, current management, and paths to personalizing therapy. Endocrinology.

[145] Heintz A.P., Odicino F., Maisonneuve P., Quinn M.A., Benedet J.L., Creasman W.T., Ngan H.Y., Pecorelli S., Beller U. (2006). Carcinoma of the ovary. FIGO 26th annual report on the results of treatment in gynecological cancer. Int. J Gynaecol. Obstet.

[146] Bristow R.E., Tomacruz R.S., Armstrong D.K., Trimble E.L., Montz FJ. (2002). Survival effect of maximal cytoreductive surgery for advanced ovarian carcinoma during paltinum era: A-meta analysis. J. Clin. Oncol..

[147] Kommoss S., Rochon J., Harter P., Heitz F., Grabowski J.P., Ewald-Riegler N., Haberstroh M., Neunhoeffer T., Barinoff J., Gomez R. (2010). Prognostic impact of additional extended surgical procedures in advanced-stage primary ovarian cancer. Ann. Surg. Oncol.

[148] Harter P., du Bois A. (2005). The role of surgery in ovarian cancer with special emphasis on cytoreductive surgery for recurrence. Curr. Opin. Oncol.

[149] Rose P.G., Nerenstone S., Brady M.F., Clarke-Pearson D., Olt G., Rubin S.C., Moore D.H., Small J.M., Gynecologic Oncology Group (2004). Secondary surgical cytoreduction for advanced ovarian carcinoma. N. Eng. J. Med..

[150] Du Bois A., Marth C., Pfisterer J., Harter P., Hilpert F., Zeimet A.G., Sehouli J. (2012). Neoadjuvant chemotherapy cannot be regarded as adequat routine therapy strategy of advanced ovarian cancer. Int. J. Gynaecol. Cancer.

[151] International Collaborative Ovarian Neoplasm Group (2002). Paclitaxel plus carboplatin versus standard chemotherapy with either single-agent carboplatin or cyclophosphamide, doxorubicin, and cisplatin in women with ovarian cancer: The ICON3 randomised trial. Lancet.

[152] Du Bois A., Lück H.J., Meier W., Adams H.P., Möbus V., Costa S., Bauknecht T., Richter B., Warm M., Schröder W. (2003). A randomized clinical trial of cisplatin/paclitaxel versus carboplatin/paclitaxel as first-line treatment of ovarian cancer. J. Natl. Cancer Inst.

[153] Du Bois A., Pfisterer J., Burchardi N., Loibl S., Huober J., Wimberger P., Burges A., Stähle A., Jackisch C., Kölbl H. (2007). Combination therapy with pegylated liposomal doxorubicin and carboplatin in gynecologic malignancies: A prospective phase II study of the Arbeitsgemeinschaft Gynäekologische Onkologie Studiengruppe Ovarialkarzinom (AGO-OVAR) and Kommission Uterus (AGO-K-Ut). Gynecol. Oncol.

[154] Du Bois A., Herrstedt J., Hardy-Bessard A.C., Müller H.H., Harter P., Kristensen G., Joly F., Huober J., Avall-Lundqvist E., Weber B. (2010). Phase III trial of carboplatin plus paclitaxel with or without gemcitabine in first-line treatment of epithelial ovarian cancer. J. Clin. Oncol.

[155] Fujiwara K., Aotani E., Hamano T., Nagao S., Yoshikawa H., Sugiyama T., Kigawa J., Aoki D., Katsumata N., Takeuchi M. (2011). A randomized Phase II/III trial of 3 weekly intraperitoneal versus intravenous carboplatin in combination with intravenous weekly dose-dense paclitaxel for newly diagnosed ovarian, fallopian tube and primary peritoneal cancer. Jpn. J. Clin. Oncol.

[156] Perren T.J., Swart A.M., Pfisterer J., Ledermann J.A., Pujade-Lauraine E., Kristensen G., Carey M.S., Beale P., Cervantes A., Kurzeder C., ICON7 Investigators (2011). A phase 3 trials of bevacicumab in ovarian cancer. N. Engl. J. Med.

[157] Tan D.S., Rothermundt C., Thomas K., Bancroft E., Eeles R., Shanley S., Ardern-Jones A., Norman A., Kaye S.B., Gore M.E. (2008). “BRCAness” syndrome in ovarian cancer: A case-control study describing the clinical features and outcome of patients with epithelial ovarian cancer associated with BRCA1 and BRCA2 mutations. J. Clin. Oncol.

[158] Huehls A.M., Wagner J.M., Huntoon C.J., Karnitz L.M. (2012). Identification of DNA repair pathways that affect the survival of ovarian cancer cells treated with a PARP inhibitor in a novel drug combination. Mol. Pharmacol.

[159] Mégnin-Chanet F., Bollet M.A., Hall J. (2010). Targeting poly(ADP-ribose)polymerase activity for cancer therapy. Cell Mol. Life Sci.

[160] Weil M.K., Chen A.P. (2012). PARP inhibitor treatment in ovarian and breast cancer. Curr. Probl. Cancer.

[161] Bryant H.E., Bryant H.E., Schultz N., Thomas H.D., Parker K.M., Flower D., Lopez E., Kyle S., Meuth M., Curtin N.J. (2005). Specific killing of BRCA2-deficient tumours with inhibitors of poly(ADP-ribose) polymerase. Nature.

[162] Farmer H., McCabe N., Lord C.J., Tutt A.N., Johnson D.A., Richardson T.B., Santarosa M., Dillon K.J., Hickson I., Knights C. (2005). Targeting the DNA repair defect in BRCA mutant cells as a therapeutic strategy. Nature.

[163] Fong P.C., Boss D.S., Yap T.A., Tutt A., Wu P., Mergui-Roelvink M., Mortimer P., Swaisland H., Lau A., O’Connor M.J. (2009). Inhibition of poly(ADP-ribose) polymerase in tumors from BRCA mutation carriers. N. Engl. J. Med.

[164] Annunziata C.M.O., Shaughnessy J. (2010). Poly (ADP-ribose) polymerase as a novel therapeutic target in cancer. Clin. Cancer Res..

[165] Chionh F., Mitchell G., Lindeman G.J., Friedlander M., Scott C.L. (2011). The role of poly adenosine diphosphate ribose polymerase inhibitors in breast and ovarian cancer: Current status and future directions. Asia Pac. J. Clin. Oncol.

[166] Patel A.G., De Lorenzo S.B., Flatten K.S., Poirier G.G., Kaufmann S.H. (2012). Failure of iniparib to inhibit poly(ADP-Ribose) polymerase *in vitro*. Clin. Cancer Res.

[167] Liu X., Shi Y., Maag D.X., Palma J.P., Patterson M.J., Ellis P.A., Surber B.W., Ready D.B., Soni N.B., Ladror U.S. (2012). Iniparib nonselectively modifies cysteine-containing proteins in tumor cells and is not a Bona Fide PARP inhibitor. Clin. Cancer Res.

[168] Fong P.C., Yap T.A., Boss D.S., Carden C.P., Mergui-Roelvink M., Gourley C., De Greve J., Lubinski J., Shanley S., Messiou C. (2010). Poly(ADP)-ribose polymerase inhibition: Frequent durable responses in BRCA carrier ovarian cancer correlating with platinum-free interval. J. Clin. Oncol.

[169] Wang Z.C., Birkbak N.J., Culhane A.C., Drapkin R., Fatima A., Tian R., Schwede M., Alsop K., Daniels K.E., Piao H. (2012). Profiles of genomic instability in hgh-grade serous ovarian cancer predict treatment outcome. Clin. Cancer Res.

[170] Mukhopadhyay A., Plummer E.R., Elattar A., Soohoo S., Uzir B., Quinn J.E., McCluggaga W.G., Maxwell P., Aneke H., Curtin N.J. (2012). Clinicopathological features of homologous recombination-deficient epithelial ovarian cancers: Sensitivity to PARP inhibitors, platinum, and survival. Cancer Res.

[171] Nishikawa H., Wu W., Koike A., Kojima R., Gomi H., Fukuda M., Ohta T. (2009). BRCA1-associated protein 1 interferes with BRCA1/BARD1 RING heterodimer activity. Cancer Res.

